# Sensors for Positron Emission Tomography Applications

**DOI:** 10.3390/s19225019

**Published:** 2019-11-17

**Authors:** Wei Jiang, Yamn Chalich, M. Jamal Deen

**Affiliations:** 1 School of Biomedical Engineering, McMaster University, Hamilton, ON L8S 4L8, Canada; jiangw35@mcmaster.ca; 2 Department of Electrical and Computer Engineering, McMaster University, Hamilton, ON L8S 4L8, Canada; chalichy@mcmaster.ca

**Keywords:** positron emission tomography (PET), photomultiplier tubes (PMT), avalanche photodiode (APD), single-photon avalanche diode (SPAD), cadmium zinc telluride (CZT), silicon photomultiplier (SiPM), digital silicon photomultiplier (dSiPM)

## Abstract

Positron emission tomography (PET) imaging is an essential tool in clinical applications for the diagnosis of diseases due to its ability to acquire functional images to help differentiate between metabolic and biological activities at the molecular level. One key limiting factor in the development of efficient and accurate PET systems is the sensor technology in the PET detector. There are generally four types of sensor technologies employed: photomultiplier tubes (PMTs), avalanche photodiodes (APDs), silicon photomultipliers (SiPMs), and cadmium zinc telluride (CZT) detectors. PMTs were widely used for PET applications in the early days due to their excellent performance metrics of high gain, low noise, and fast timing. However, the fragility and bulkiness of the PMT glass tubes, high operating voltage, and sensitivity to magnetic fields ultimately limit this technology for future cost-effective and multi-modal systems. As a result, solid-state photodetectors like the APD, SiPM, and CZT detectors, and their applications for PET systems, have attracted lots of research interest, especially owing to the continual advancements in the semiconductor fabrication process. In this review, we study and discuss the operating principles, key performance parameters, and PET applications for each type of sensor technology with an emphasis on SiPM and CZT detectors—the two most promising types of sensors for future PET systems. We also present the sensor technologies used in commercially available state-of-the-art PET systems. Finally, the strengths and weaknesses of these four types of sensors are compared and the research challenges of SiPM and CZT detectors are discussed and summarized.

## 1. Introduction

Medical imaging plays a very important role in the clinical analysis and diagnosis of diseases by providing visual representations of the interior structures of a subject and/or the physiological processes hidden underneath the skin. Medical imaging also helps to establish a database with thousands of anatomical and physiological images. The emergence of these databases become a powerful tool for training both doctors and emerging machine-based systems to identify abnormalities. This resource will become more beneficial considering the significant breakthroughs developing in the fields of big data and machine learning [1]. 

The field of medical imaging has employed many imaging techniques besides positron emission tomography (PET), the most notable being X-ray computed tomography (CT), magnetic resonance imaging (MRI), ultrasound imaging (UI), and optical coherent tomography (OCT). Among these imaging techniques, PET has become one of the most powerful tools to acquire functional images due to its high sensitivity to differences in the metabolic and biological activities at the molecular level [2]. PET is currently being used in a wide variety of clinical areas, such as oncology for cancer or tumor diagnosis and staging [2], neurology for Alzheimer’s disease and movement disorders [3], and cardiology in coronary artery disease and myocardial viability assessment [4]. 

In recent years, PET has also become an important tool in preclinical applications where animal models are used in place of humans to study disease and experiment with new drug development and treatment strategies. While animal PET imaging is mostly for small rodents, there have also been studies done on primates due to the high homology of genes with humans [5]. PET, as a molecular imaging technique, can offer unique non-invasive and in vivo imaging beneficial to the study of the biological and biochemical process of the subjects during the experiments with animal models. As a result, a variety of animal PET scanners have been designed and developed for preclinical applications [6,7,8,9,10]. 

PET has also been integrated with other techniques like CT and MRI to develop multimodal imaging systems that take the advantages of combining both functional and anatomical images for improved diagnostics. While the integration of PET and CT has already seen widespread adoption, the PET/MRI systems took longer to develop owing to the photomultiplier tube (PMT) detector limitations of early PET systems. The integration of PET/MRI requires that the PET system must be compatible with the high magnetic fields from an MRI system. At the same time, the MRI image should not be degraded by the PET system due to the extra noise and electrical interference. Thanks to the advances of photo-sensor technologies in recent years, the PET/MRI system was made commercially available and used in many clinical applications [11,12,13,14,15,16,17].

It became clear that the selection and optimization of the sensor technology in the development of PET detectors was vital to the overall price, performance, and integration. For instance, while PMTs were initially selected for the development of PET systems because of their high gain and low noise, their fragility, bulkiness, high operating voltage, and magnetic field sensitivity led to intensive research on solid-state photodetectors like avalanche photodiodes (APDs), silicon photomultipliers (SiPMs), and their potential for PET applications. Moreover, the direct-conversion semiconductor material cadmium zinc telluride (CZT) also garnered interest for PET applications due to the benefit of removing the limitations associated with scintillators such as price and complexity of the assembly of scintillators onto the photodetectors.

In this review, we studied four types of photodetectors for PET applications: PMTs, APDs, SiPMs, and CZT detectors, with special focus on SiPMs and CZT detectors. The physics of PET and time-of-flight (ToF) PET are introduced in Section 2. Different types of PMTs and their PET application are stated and summarized in Section 3. A brief discussion about APD for PET is presented in Section 4, which is followed by a detailed review of SiPMs and their applications in Section 5. The CZT detectors for PET applications are discussed and compared in Section 6. In Section 7, the state-of-the-art and commercially available PET systems are described. Finally, the paper is concluded with a comparison of the features of these four types of sensors and a discussion of the key research challenges and future direction of the two promising photosensors for PET applications in Section 8. 

## 2. Positron Emission Tomography

### 2.1. Physics of Positron Emission Tomography

The basic principle for PET is the coincident detection of a pair of gamma rays generated from the annihilation events of the positrons from the radioactive tracer injected into a subject. The radiotracers for PET applications are analogous to common biological molecules such as glucose, peptide, and proteins, in which a radioisotope is used to substitute one of the constituents of the tracer [18,19]. For example, ^18^F is used to replace the ^16^O of glucose to produce ^18^F-fluorodeoxyglucose (FDG), which is analogous to glucose and can indicate levels of cellular metabolism. Another widely used tracer is ^11^C-L-methionine, analogous to the amino acid, which can be used as an indicator of cancer malignancy based on the utilization of the amino acid [18].

**Figure 1:**
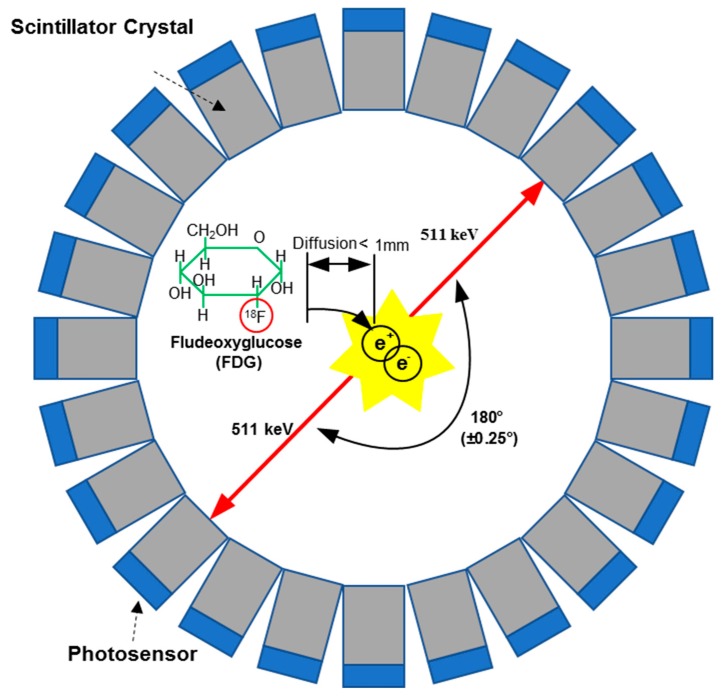
The basic principle of a positron emission tomography (PET) system: A PET detector ring detects a pair of gamma photons with an energy of 511 keV (red arrows) which results from the annihilation of an electron with a positron emitted by the radiotracer (FDG).

**Figure 2:**
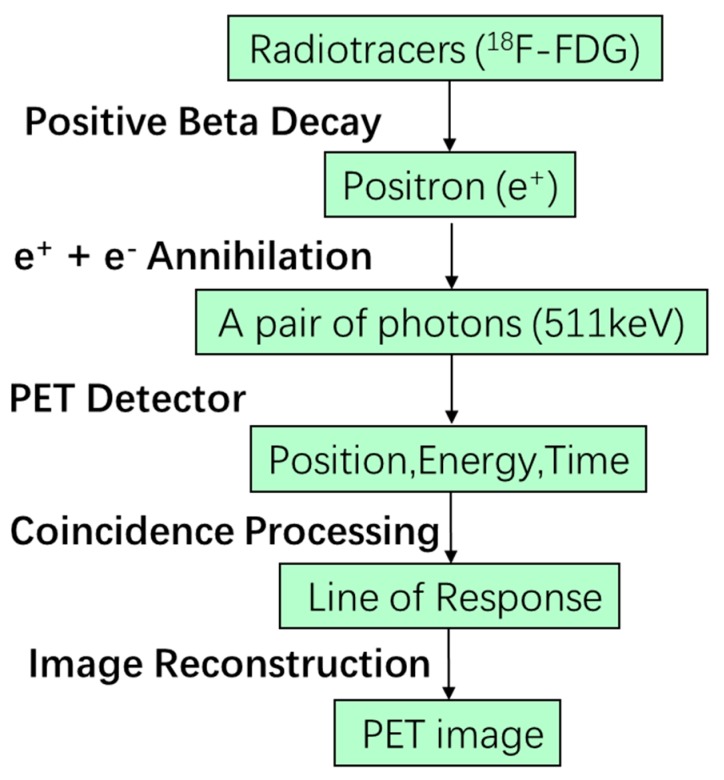
Detection flow of a positron emission tomography (PET) system.

The illustration of a general PET imaging system is shown in Figure 1 [20]. The first step of the PET image scanning is the injection of a radioactive tracer into the subject. The radioactive tracer arrives at the targeted organs or tissues through the circulatory system after a certain amount of time and participates in the metabolic process of the subject. Since the radioisotopes in the tracer are not stable, they decay (i.e., through β+ decay) during the metabolic process with a specific half-life decay time (around 110 minutes for ^18^F and 20 minutes for ^11^C). During the decay process, positrons are generated and travel to collide with the electrons of the neighboring atoms in an annihilation process. The annihilation generates two gamma rays with an energy of 511 keV and a separation of approximately 180 degrees [21]. In order to detect the gamma rays due to the annihilations, scintillation crystals are used to absorb and convert the high energy gamma rays into low energy visible photons. One of the most commonly used scintillators is lutetium-(yttrium) oxyorthosilicate (L(Y)SO) due to its high density, high light output, fast decay time, and excellent energy resolution. Then, a photosensor like a PMT, APD, or SiPM is used to convert the light signal into an electrical signal. The scintillators, detectors, and the readout electronics are generally assembled together to form a modular detector, which are used to build a detecting ring to record three electronic signals: the time when the gamma ray hits the detector, the position where the gamma ray hits the detector, and the energy of the gamma ray [19]. These electronic signals are then processed through the coincidence unit to get the true events generated by the same annihilation which occur along the line of response (LoR). Finally, the raw data of thousands of LoRs are used to generate the PET image through image corrections and reconstructions. The entire detection flow is shown in Figure 2.

### 2.2. Time-of-Flight (ToF) PET

**Figure 3:**
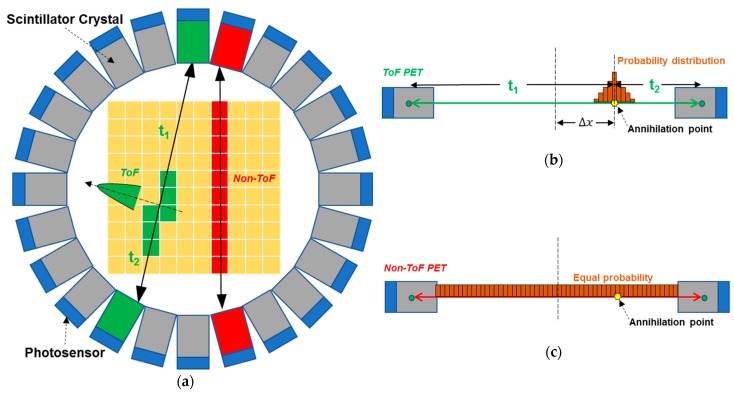
Concept of time-of-flight positron emission tomography (ToF PET): (**a**) Illustration of a detector ring detecting pairs of gamma photons from the annihilation events with (green) and without (red) the ToF technique; (**b**) The probability distribution of the annihilation position along the line of response (LoR) in ToF PET; (**c**) The equal probability of the annihilation position along the LoR in non-ToF PET.

Due to improvements in the timing resolution performance of photosensors, the time-of-flight (ToF) technique can be applied to the conventional PET to significantly increase the sensitivity and signal-to-noise ratio (SNR), thus improving the image quality [18,19]. Figure 3 shows the concept for ToF PET and its comparison to the conventional PET. In the conventional PET, the time difference between a pair of gamma rays generated from the annihilation event can only be used to find the LoR by the coincidence processing unit with the coincidence window usually set to 3–5 ns. Once a LoR is determined, we only know that the annihilation occurred along this LoR. The probabilities for all the voxels along this LoR to locate the position of the annihilation event are the same, as shown in Figure 3a,c. Therefore, in the conventional PET, the noise from all voxels will be accumulated to reduce the system SNR, resulting in degraded image contrast and resolution [22,23]. However, due to the higher timing resolution in ToF PET, the location of the annihilation event can be located by measuring the difference of the arrival time of each photon at two detectors along the LoR. The uncertainty of location for the annihilation point due to the time jitter is limited to a short range along the LoR, as shown in Figure 3b.

Compared to the conventional PET, the improvement of SNR in ToF PET is given by:

(1) $$\frac{SNR_{ToF}}{SNR_{Non-ToF}}=\sqrt{\frac{2D}{c\times \Delta t}},$$

(2) $$G=\frac{2D}{c\times \Delta t},$$

where the D is the diameter of the object being scanned, c is the speed of light in vacuum, Δt is the variation of arrival time, defined as the coincidence resolving time (CRT), and G is the gain of effective sensitivity [24]. For example, if the system is assumed to achieve a CRT of 300 ps, and the diameter of the subject is 40 cm, we can calculate that SNR will increase ~3 times and the effective sensitivity will increase ~9 times. The increased SNR and sensitivity will contribute to the improvement of the SNR of the final PET image. One comparison of the image quality between ToF PET and non-ToF PET was reported in [25], which showed three clinical images from a patient with colon cancer by using CT, non-ToF PET and ToF PET. The lesion for colon cancer was easily detected by the ToF PET but could not be observed in the non-ToF PET.

### 2.3. PET Detector

**Figure 4:**
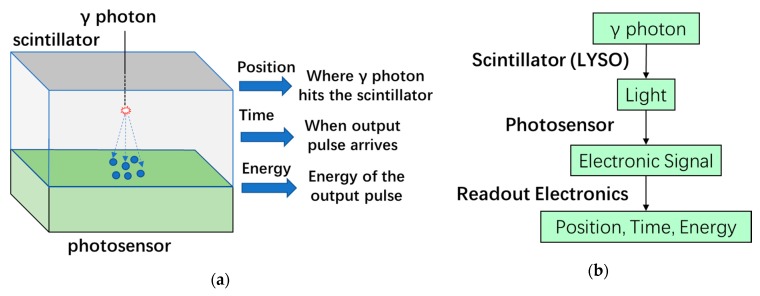
Positron emission tomography (PET) detector: (**a**) Structure of a PET detector; (**b**) Detection flow chart of a PET detector.

The basic structure of a PET detector is shown in Figure 4a, which mainly consists of a scintillator block mounted on a photosensor. The main function of a PET detector is to acquire three types of information: the position where the gamma photon impacts the scintillator, the time when the output pulse from the photosensor arrives, and the energy of the output pulse. The photon detection flow is illustrated in Figure 4b.

The photosensor in a PET detector plays a very important role in system performance in terms of spatial resolution, coincidence timing resolution and energy resolution. There are generally four types of photodetectors being employed in research and commercial PET systems: PMTs, APDs, SiPMs, and CZT detectors. Unlike the former three sensors which need a scintillator to convert the high-energy gamma ray to low-energy visible light, CZT detectors can generate an electronic signal by directly detecting the gamma photon. Among these four photosensors, PMTs were the first to be employed in the design of PET detectors due to their favorable qualities such as high gain, low noise, and high timing performance. In Section 3, a comprehensive description on the operating principles, key performance parameters, and PET applications of PMTs is presented.

## 3. Photomultiplier Tubes (PMTs)

### 3.1. Operating Principles of PMTs

**Figure 5:**
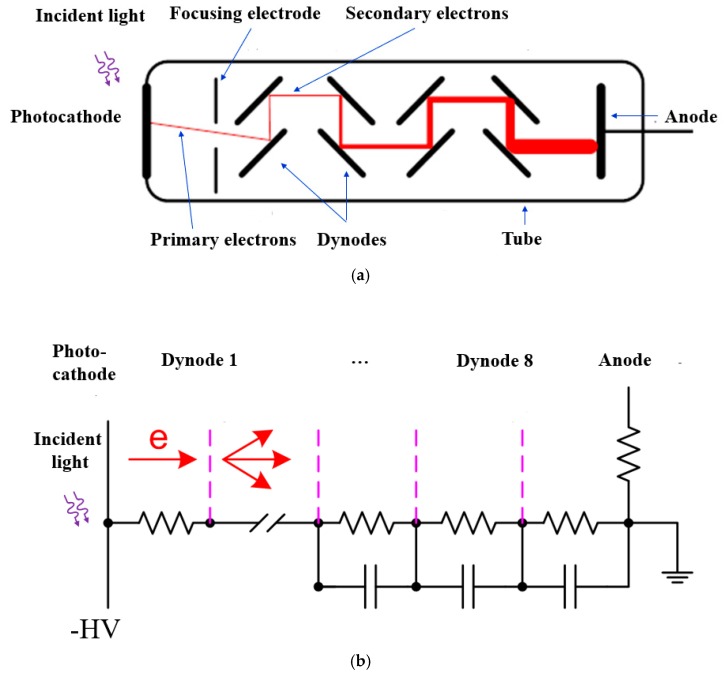
Principle of the photomultiplier tube (PMT): (**a**) Simplified conventional structure; (**b**) Simplified high-voltage biasing network.

The schematic diagram of a typical PMT structure is illustrated in Figure 5 [26]. The primary components of a PMT are a photocathode, a series of electrodes (dynodes), and an anode, which are enclosed in a vacuum glass tube. The different electrodes are biased at progressively higher voltages to form increasingly higher internal electric fields. When the incident photons hit the photocathode through the glass window, photoelectrons are generated from the photosensitive material on the photocathode due to the photoelectric effect. The high internal electric field between the photocathode and the first dynode significantly increases the kinetic energy of the photoelectrons allowing them to generate a secondary emission of electrons upon colliding with the dynode. This process is amplified at each progressive dynode as the electrons accelerate between them. Eventually, the anode collects a large number of electrons to generate a high photo-current pulse, which is significantly greater than the noise current. With proper design of the photocathode and the multiple stages of dynodes, PMTs can easily achieve multiplication gains of 10^6^ [27].

**Figure 6:**
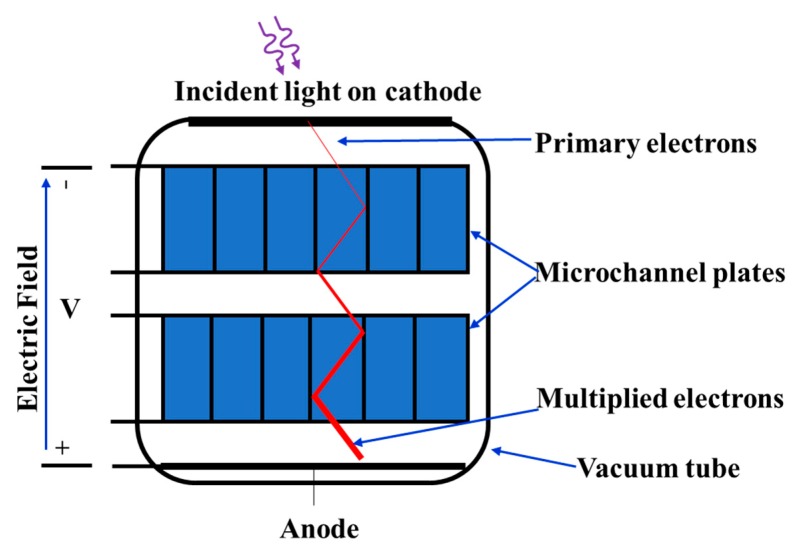
Illustration of a microchannel plate photomultiplier tube (MCP-PMT) (the microchannels are usually made of highly resistive materials (e.g., lead glass cladding) with the inner wall coated by high secondary emission materials such as MgO and Al_2_O_3_).

There also exists microchannel plate (MCP) PMTs, shown in Figure 6, that utilize the gain effect of the secondary electron emissions. Instead of using a chain of separate dynodes, MCP-PMTs are composed of a large number of micron-sized channels coated with a conductive emissive dynode material. A high electric field along the channel is present when a high voltage is applied. When a high energy photoelectron emitted by the photocathode enters the channel, it is absorbed and causes secondary emissions from the coating material of the internal walls. This process is repeated by subsequent emissions along the channel to result in an exponentially increased number of electrons at the anode to generate a photocurrent distinguishable from the noise. Typical diameters of the channel are 3–10 μm. Higher gain can be achieved by arranging two or three microchannel plates in series. The first microchannel plate can be placed very near to the cathode, resulting in a high performance for the transit-time spread, which can be as short as 25 ps [26].

**Figure 7:**
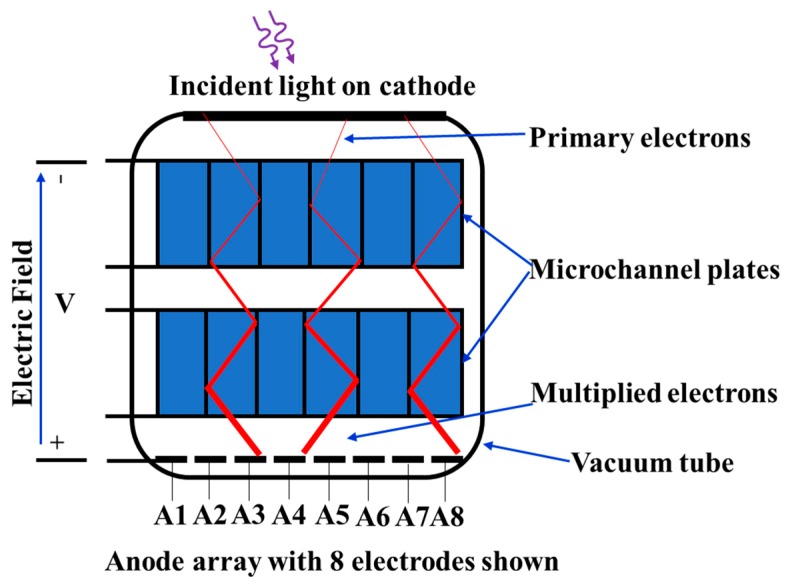
Structure of a position-sensitive photomultiplier tube (PS-PMT) with microchannel plates.

By replacing a single anode plate with an array of separated anodes, the individually detected pulses can give position sensitivity of the incident photons on the photocathode. PMT configurations with multiple anode outputs like this are thus called position-sensitive (PS) PMTs. Figure 7 shows one typical structure of the PS-PMT. 

**Figure 8:**
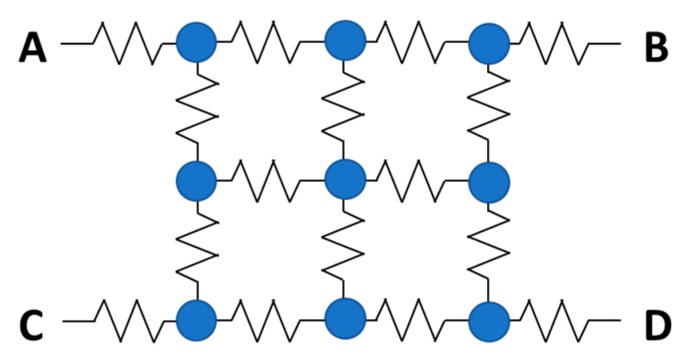
Resistive network for a 3 × 3 anode array (each blue dot represents one signal anode).

In the PS-PMT, if the pulses from the multiple anode array are processed individually, then multiple channels of readout electronics are required, which increases the complexity, power consumption and cost. In order to reduce the number of output channels, a resistive network can be employed to connect multiple anodes. Figure 8 shows an example to reduce a 3 × 3 anode array to a 2 × 2 output array. Based on the four output signals at A, B, C, and D from the resistive network, the position information can be calculated using Anger logic as follows:

(3) $$X=\frac{B+D-\left(A+C\right)}{A+B+C+D}\;Y=\frac{A+B-\left(C+D\right)}{A+B+C+D}.$$

Here (X, Y) are the calculated coordinates of the position where the incident light struck the photocathode with the assumption that the origin is the center of the photocathode plate.

### 3.2. Key Performance Parameters

Ideally, if there is no single photon absorbed by the photocathode, there will be no output pulse from the anode of the PMT. However, electrons randomly emitted from the photocathode and dynodes by the process of thermionic emission and/or field emission generate output pulses even if the PMT is kept in the dark. These “dark” pulses per unit time are termed the dark count rate (DCR) of the PMT. The DCR of the PMT is mainly determined by the cathode material and the design of the dynode chain. Compared to a solid-state sensor, the vacuum environment enables a PMT to achieve a relatively low DCR on the order of tens of counts per second [28].

To describe the timing characteristics of a PMT, the electron transit time response (TTR) and the transit time spread (TTS) are used. The TTR is the average time difference between the arrival of a photon at the photocathode and the output pulse at the anode. The TTS is the standard deviation of the transit time distribution, also known as timing resolution or timing jitter [28]. Furthermore, the TTS is the critical parameter for PET applications since it represents the uncertainty of the photon arrival time and has an impact on CRT.

Photon detection efficiency (PDE) of a PMT is determined by the collection efficiency and the internal quantum efficiency (QE) of the photocathode material, which is used to describe the probability of emission of a photoelectron per incident photon. It is usually measured as a ratio between the number of generated photoelectrons and the number of incident photons. The PMT’s quantum efficiency is wavelength dependent and has a typical value of ~25% [27]. Due to the limitation of collection efficiency, the PDE is smaller than the QE since not every photoelectron can be collected to generate a detectable pulse.

### 3.3. PET Applications

The first commercially available PET imaging systems were based on the PMT technology. For the last two decades, the spatial resolution of the conventional human PET system was usually 4.5–6 mm due to the limitation of the sensing technology. In order to improve resolution, more and more PMTs with fast response time and lower TTS were fabricated and investigated for the potential to employ ToF techniques.

A ToF PET detector consisting of a flat panel PS-PMT (Hamamatsu R8400-00-M64 MOD) and a 16 × 16 array of LYSO crystals of size 2.9 × 2.9 × 20 mm^3^ was developed and characterized in [29]. The PS-PMT had 64 output anodes (8 × 8 array), which connected to a 64-channel readout application-specific integrated circuit (ASIC). The detector was evaluated in ToF PET applications and achieved an average full width at half maximum (FWHM) energy resolution of 10.9% at 511 keV. By using a BaF_2_ reference detector, experiments showed that the detector had an average FWHM timing resolution of 505 ps. Two other fast PMTs—the R9800 (Hamamatsu) and R11194 (Hamamatsu)—were evaluated and investigated for ToF PET applications [30]. A LYSO crystal of size 4 × 4 × 10 mm^3^ was coupled with the PMTs as a test detector. A ^22^Na point source was placed in the center between the test detector and a reference detector having a timing resolution of 200 ps. By employing an optimal constant-fraction discriminator (CFD) setup with 1.0 ns CFD delay, the average FWHM CRT of seven PMT samples was 220.5 ps for R9800 and 227.8 ps for R11194. The average FWHM energy resolution was 11.1% for R9800 and 11.8% for R11194.

Besides the conventional PET detector, in which one PMT is only used for decoding one crystal block, a PET detector using a PMT-Quadrant-Sharing (PQS) configuration was developed [31,32,33]. In the PQS configuration, only a quadrant of the PMT was used to decode the crystal block, resulting in higher decoding resolution (the ratio of numbers of crystals in the whole system to the numbers of PMTs in the whole system). A human PET based on PMTs with a diameter 38 mm and LYSO scintillator array with a pitch of 2.4 mm gave a decoding resolution of 256 [31]. In [32], the initial results of the timing performance of a PET detector utilizing a PQS configuration was presented. Here, a 13 × 13 LYSO array of size 4 × 4 × 20 mm^3^ was coupled to four fast Hamamatsu R9779 PMTs by means of optical grease. A single LYSO of 4 × 4 × 20 mm^3^ mounted on a R9779 PMT was employed as a reference detector in the coincidence measurement setup with a ^22^Na point source placed close to the reference. The system achieved a FWHM CRT of 432 ps. Another PET detector made by coupling a 13 × 13 LYSO array of small size (1.4 × 1.4 × 10 mm^3^) onto a small Photonis XP1912 PMT was measured to achieve a FWHM CRT of 551 ps. A whole PET system for human imaging using the PQS configuration was reported in [33]. The detector ring was made up of 24 detector panels with an axial field-of-view (FoV) of 27.6 cm and a ring diameter of 87 cm. Each panel consisted of 3 × 7 detector blocks, and each block contained an array of 16 × 16 LYSO crystals with a size of 2.35 × 2.35 × 15.2 mm^3^. The whole system used 129,024 crystals and 576 PMTs. The system performance was measured by placing a ^68^Ge line source at the center of the PET ring. It achieved an averaged FWHM energy resolution of 11.2% and FWHM CRT of 473 ps. By using the 2D filtered back-projection (FBP) method, the system demonstrated a spatial resolution of 2.87 mm, which was improved to 1.55 mm when applying 3D-iterative reconstruction method with a point spread function. Table 1 presents the summary and comparison of some commercial PMTs used in PET. By using the LYSO or LGSO (Lu_2x_Gd_2(1-x)_SiO_5_:Ce) crystals, commercial PMTs have achieved a CRTs between 200 and 500 ps FWHM and an energy resolution of ~11% FWHM.

Although the PMT can achieve high gain, relatively low noise, high timing resolution and reasonable QE, it has some important disadvantages. PMTs are fragile and bulky, sensitive to mechanical vibrations and electromagnetic disturbances, require high operational voltages (>1 kV) and are costly. These disadvantages severely limit their practical use in compact and portable instruments and other applications which have large magnetic fields or require low-cost, low-voltage detectors. For these reasons, solid-state solutions like APDs and SiPMs have been proposed to replace PMTs in many applications.

## 4. Avalanche Photodiodes (APDs)

### 4.1. Operating Principles of APDs

**Figure 9:**
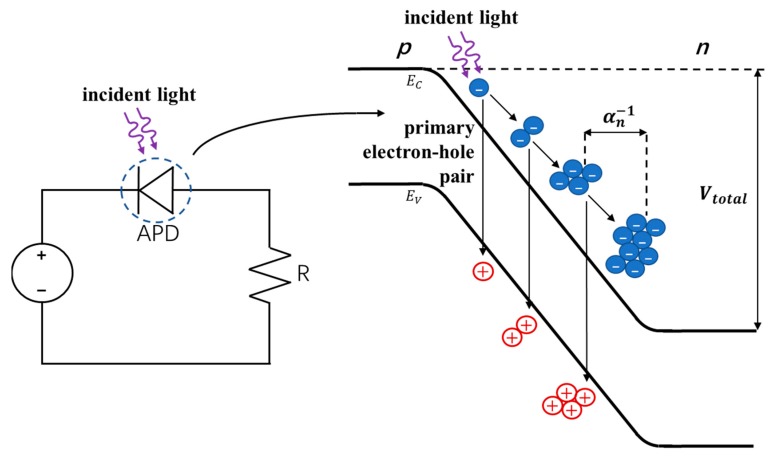
The impact ionization process. Here, $\alpha_{n}^{-1}$ is the average distance between each electron multiplication event, while $V_{total}$ is the applied reverse bias plus the built-in potential.

APDs are structurally similar to p-n or p-i-n photodiodes, but the gain mechanism is from avalanche multiplication. They are biased at a large reverse voltage such that when an incident photon generates a carrier within the depletion (space-charge) region, it is accelerated due to the high electric field present. With enough energy from the electric field, the carrier can generate an electron-hole (e-h) pair through impact ionization when it collides with the lattice, as illustrated in Figure 9. This process can be repeated by the initial carrier and subsequently generated carriers through avalanches, resulting in an appreciable increase in the detectable current. The impact ionization coefficient α for the main charge carrier (electrons in the case presented in Figure 9 for APDs) determines the multiplication gain and represents the number of e-h pairs generated per unit length by the carrier. Due to the stochastic nature of the avalanche effect, there exists some variation in the multiplication gain since not every injected or photogenerated carrier leads to the same multiplication. This noise is measured by the excess noise factor *F* and is dependent on the ratio k of the ionization coefficients for electrons ($\alpha_{n}$) and holes ($\alpha_{p}$) of the material used. When considering electron injection/multiplication in APDs, $k=\alpha_{p}/\alpha_{n}$ ($\alpha_{n}/\alpha_{p}$ for hole injection/multiplication) and k should be minimized to reduce this noise. The k value is 0.02 for Si and ~0.5 for Ge and III-V compound semiconductors and is an important reason why the majority of commercial APDs are made of Si [36] to reduce excess noise.

### 4.2. Key Performance Parameters of APDs

The performance of an APD is primarily specified in terms of its spectral response, quantum efficiency, gain, and dark current. The spectral response, or responsivity, corresponds to the generated current (A) per unit power (W) of light incident on the APD for different wavelengths. The quantum efficiency is also a function of wavelength and indicates the percentage of photons that reach the depletion region and trigger an avalanche. The quantum efficiency and responsivity are dependent on the light transmitted into the semiconductor, the percentage of photons absorbed and converted to carriers in the multiplication process, and the current collected from non-recombined carriers. The (multiplication) gain described above through the impact ionization process is ideally sought to be as large and stable as possible to more accurately identify low-level incident light. Multiplication also applies to dark current that primarily occurs from thermally generated e-h pairs, and the random thermal motion and fluctuations of charge carriers. Together, low dark current and high gain allows for distinguishability between the amplified noise and amplified light-triggered events. The dark current and gain vary with reverse bias voltage and temperature such that APDs can be set to function at some optimal operating point to maximize the SNR. While APDs can have gains up to around 1000 [27], Si APDs, such as those from Hamamatsu, typically have an internal gain around 50 at the required 420 nm wavelength for PET [37].

### 4.3. PET Applications

For PET applications, multiple tests using APD detectors were performed [38,39,40,41,42,43], showing increased acceptance and support for APD-based scanners over PMTs moving forward. Although the gain of APDs was not as good as in PMTs, they offered more benefits such as reduced cost, smaller size, and insensitivity to magnetic fields. This allowed for significantly easier integration into multi-modal systems such as with MRI, and opened opportunities for combined PET/MRI systems. For instance, the PET scanner in the latest PET/MRI system from Siemens (Biograph mMR) is based on APD technology (more details about this system are provided in Section 7.2). It is important to note that as APD performance improved and its size became smaller, they would offer lower noise and capacitance, thus improving energy and timing resolution. Finer pixel pitch would also result in improved spatial resolution. In Table 2, we summarize some of the APDs tested for PET and their respective results, followed by a more detailed discussion.

In 2005, a light-sharing technique was tested to minimize the number of photodetectors and electronic channels required for PET imaging [38]. Here, multiple scintillators were coupled to one APD instead of a one-to-one configuration. An initial performance study was done with a single APD coupled to a chemically etched 4 × 4 × 10 mm^3^ Teflon-wrapped LSO crystal and a custom ASIC preamplifier. The CRT against a plastic scintillator coupled to a PMT was 870 ps FWHM, with an energy resolution of 12.1% FWHM. Following this, a 9 × 9 crystal array of half the size was then coupled to a 2 × 2 array of the APDs showing promising results of 20.9% energy resolution and 2.47 ns timing resolution FWHM. Another Hamamatsu detector (S8550) with improved gain and QE at 420 nm was compared against previous versions and the S8664-55 as a common PET/CT detector showing improved timing (CRT ~2.15 ns) and energy (12.8%) resolutions, as listed in Table 2 above [39].

Then in 2007, there was a performance evaluation of LabPET, the first commercial APD-based PET scanner for the imaging of small animals [40]. Each detector of the scanner was made of a phoswich pair of LYSO/LGSO scintillators (each 4 × 2 × 10/12 mm^3^), optically coupled along one long side and read by a single APD on a 55° wedge. An FBP reconstructed resolution of 1.3 mm (tangential) and 1.4 mm (radial) was obtained at the FoV center, and a maximum likelihood estimation method (MLEM) reconstruction of a micro-resolution phantom showed clear distinction between 1.35 mm spots with fair identification of 1 mm spots. Development of the LabPET II APD detector module soon followed which was capable of achieving submillimeter spatial resolution [44]. It contained two monolithic 4 × 8 APD pixel arrays (each 1.1 × 1.1 mm^2^ active area at 1.2 mm pitch) coupled to an 8 × 8 LYSO scintillator array for direct one-to-one coupling. The 64-channel APD array allowed for individual pixel readout for parallel signal processing. This module was later proposed as a building block for merged dual-modality PET/CT scanners, with improvements such as a QE of 60% at 420 nm (previously 40%) and the use of unbound specular reflective film between pixels to improve total internal reflection [41]. It achieved an intrinsic spatial resolution of 0.81 ± 0.04 mm and time resolution of 3.6 ± 0.3 ns FWHM, with a mean energy resolution of 20 ± 1% among all the pixels for PET.

APD experiments were also carried out in unique configurations such as having the annihilation radiation enter the scintillator edge-on using position-sensitive APDs (PS-APDs) with the necessary crystal segmentation to record all three spatial coordinates simultaneously [45]. This allowed >90% light collection efficiency independent of the interaction location, and the photon depth-of-interaction (DOI), which represents the position inside the crystal where the gamma ray interacts with the crystal lattice, could be directly measured by the longitudinal segmentation. These PS-APDs, manufactured by RMD Inc., had a gain of 1000, 77% QE at 400 nm, and were operated around –1750 V. The 0.91 × 0.91 × 1 mm^3^ LYSO crystals were arranged in an 8 × 8 array to match the 8 × 8 mm^2^ active area of a PS-APD. This research led to the proposal and development of a breast-dedicated PET camera with an initial prototype containing a total of 512 PS-APDs and 32,568 crystals in 2013 [42]. It achieved a spatial resolution of 0.84 ± 0.02 mm with energy and time resolutions of 10.62 ± 0.04% and 15.7 ± 0.2 ns FWHM, respectively. Some challenges that arose from these tightly packed APD arrays were the variation in gain and leakage current with respect to bias voltage and temperature, with the gain variation being reported as 6%/°C at 18 °C for the above PS-APDs [46]. Thermoelectric Peltiers were used and cooled by chilled water regulated heatsinks to hold the temperature to within 0.67 °C (18.20 °C to 18.87 °C) over 7.5 hours under steady state operating conditions.

Another such organ-specific project was a brain PET [43] insert for existing MRI equipment. Compared to dual-modality PET/CT systems, PET/MRI systems can reduce the risk of ionizing radiation, provide better soft tissue contrast of grey and white matter, and correct for attenuation and scattering of PET images [43]. The building block of this scanner consisted of large, dual layer monolithic LYSO:Ce scintillators of trapezoidal shape coupled to a pair of Hamamatsu S8550-02 APD arrays. The scanner consisted of 208 of these blocks, split into four rings of 52 each. It achieved an energy resolution of 13.2% FWHM, timing resolution of 27 ns FWHM, and spatial resolution as good as 2.1 mm FWHM for ^22^Na using neural networks position determining algorithms. The timing resolution was reported to improve to a few nanoseconds with updated front-end electronics.

This same group also ran Geant4 (GEometry ANd Tracking)-based simulations comparing the performance of APD arrays (Hamamatsu S8550-02) with that of Silicon Photo-Multipliers (SiPMs) (SensL Array2) and Hamamatsu’s equivalent named the Multi-Pixel Photon Counter (MPPC) (S11828-344M) [47]. The building block of these SiPM arrays are single-photon avalanche diodes (SPADs). They concluded that SiPMs with high photon detection efficiency could replace APDs with regards to spatial resolution and linearity, and offer other benefits such as excellent timing resolution below 1 ns, and ~10^6^ intrinsic gain equivalent to that of PMTs, allowing single photon detection that APDs were incapable of due to their limited gain. Eventually, SPADs started to be used instead of APDs and allowed operating voltage bias to lower into the tens of volts using standard silicon CMOS (complementary metal-oxide-semiconductor) processes.

## 5. Silicon Photomultipliers (SiPMs)

An array of parallelly-connected SPADs is called an analog SiPM. The output of an analog SiPM is the sum of the individual currents of all SPAD pixel cells. The output amplitude of the current of an analog SiPM is proportional to the number of SPAD breakdowns, be it from photons or noise. As the basic photon-detecting unit, the performance of the single SPAD pixel has great influences on the performance of the array. Therefore, in this section the SiPM cell, or SPAD, is first discussed, then followed by a study on SiPMs.

### 5.1. Operating Principles of SPADs

**Figure 10:**
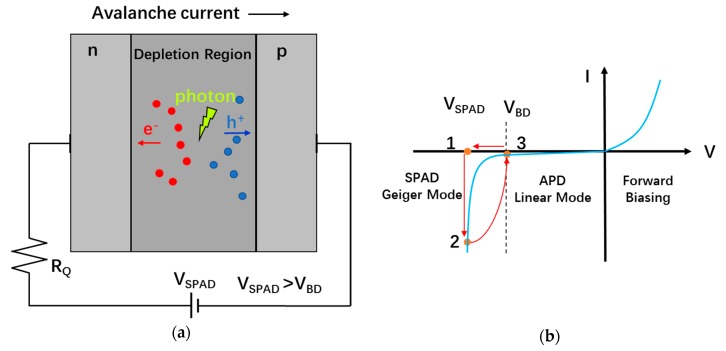
Principle of single-photon avalanche diode (SPAD) operation: (**a**) Avalanche breakdown process in a reverse biased p-n junction; (**b**) I-V characteristic representation of SPAD operation.

A SPAD is a p-n junction that is reverse-biased at an excess voltage *V_EX_* above the breakdown voltage *V_BR_* so as to operate in the Geiger mode (in analogy to a Geiger–Müller detector). Geiger-mode operation creates a large enough electric field across the depletion region of the p-n junction so that the impact ionization process triggered by an initial photogenerated charge carrier is self-sustaining [25,48]. This avalanching process can generate a large internal current flow from only a single photon, depicted in Figure 10a. Figure 10b illustrates the operating states of a SPAD. Initially, when reverse biased above breakdown, the SPAD stays in an OFF state (1) where there is no triggering event to initiate avalanching. When a charge (generated by photon absorption or other means) enters the depletion region and triggers avalanching, the SPAD is brought into its high-current ON state (2). Due to the existence of a large quenching resistor $R_{Q}$, the initiated avalanche causes the voltage across the depletion region to be reduced, bringing the SPAD to point (3), where the voltage is below the breakdown voltage. Here, the avalanche process is no longer self-sustaining and is quenched. The voltage drop can be sensed by a fast discriminator to generate a digital pulse which represents the photon arrival time if the first charge was a photon-generated charge [49,50]. After the quench of the avalanche, the SPAD voltage is recharged back to initial biasing conditions of *V_SPAD_*. 

### 5.2. Key Performance Parameters of SPADs

In order to ensure Geiger-mode operation, the breakdown voltage of the SPAD junction should first be measured. The breakdown voltage depends on both the p-n junctions biased for photon detection and the fabrication details of the CMOS process used for the design. It is generally known that the breakdown voltage of SPADs become lower with the scaling down of CMOS technology because of the increased doping concentrations and reduced junction widths that come with each new generation. Another important parameter for evaluating the performance of SPADs is the temperature coefficient of the breakdown voltage. The breakdown voltage increases with temperature because the increased photon scattering at higher temperatures make it more difficult for the electrons or holes to meet the energy threshold for avalanche [51,52]. The breakdown voltage temperature coefficient can vary from several to tens of millivolts per degree Celsius depending on the materials used, doping concentration and diode structure. For example, a SPAD (p+/n-well junction) designed in a 180 nm CMOS process was measured to have a breakdown voltage temperature coefficient of 7.14 mV/°C [53]. Another SPAD using the n+/p-well junction based on a 130 nm CMOS process showed a similar breakdown voltage temperature coefficient of 7.22 mV/°C [54,55]. SPADs based on the p-well/buried deep n-well (DNW) junction were reported to have a higher breakdown voltage at room temperature of 20 V (180 nm process) in [56] and 12.4 V (130 nm process) in [57], with higher temperature coefficients of 40 mV/°C and 20 mV/°C respectively. 

Since a SPAD’s gain is theoretically infinite, it operates digitally by counting incident photons instead of outputting an amplified current such as with APDs. Therefore, like PMTs, the SPAD’s noise performance is characterized by a DCR, defined as the avalanche pulses per second when there is no illumination. There are several carrier generation mechanisms responsible for the DCR, such as generation-recombination, band-to-band tunneling and trap-assisted tunneling. Among these noise generation mechanisms, the main mechanism at room temperature is generation-recombination, where thermally generated free carriers are created within a diffusion length of the depletion region of the SPAD [58,59]. Therefore, the DCR will increase with the operating temperature. Moreover, for a SPAD fabricated in the CMOS process, there exists higher level of impurities and defects which induce many energy levels in the forbidden band. This increases the probability of tunneling and thus leads to a higher DCR. Higher excess bias voltages also increase DCR since a higher electric field across the SPAD junction not only increases the likelihood of avalanche by photon detection, but also by noise sources. The DCR for current SPADs fabricated in CMOS technology can be optimized down to tens of Hz at room temperature [60,61].

A performance parameter associated with DCR is the afterpulsing probability (AP). That is, the probability of secondary avalanches caused by the emission of carriers trapped during the primary avalanche. The defects and impurities in the depletion region tend to trap some carriers generated during the avalanche process and these trapped carriers can be released later at random time intervals [62,63]. If the biasing voltage of the SPAD is completely recharged before all the trapped carriers in the trap centers have been released, a secondary avalanche might occur. The most efficient way to lower the AP is to optimize the SPAD front-end circuits. Typically, there are two main types of front-end circuits: passive quench and reset (PQR) and active quench and reset (AQR). A time-gating technique in [54] is also used to reduce the AP of a SPAD implemented in a 130 nm CMOS process. It was shown that with proper design of quenching and reset circuits, an AP of <1% can be achieved [64,65].

The photosensitivity of a SPAD is defined by its PDE, which is the ratio of the number of detected photons to the number of total incident photons. The PDE is the product of the geometric fill-factor (FF) (the ratio of photo-sensitive area to total pixel area), absorption probability (wavelength dependent), and avalanche triggering probability [66]. The SPADs implemented in standard silicon CMOS technology usually suffer from a low PDE due to two main reasons: the photon reflection and absorption of a thick passivation layer and the internal dielectric oxide insulation layers, and a thin depletion region. 

Lastly, there is the timing jitter of the SPAD. It is the variation in the delay between the output pulse of the SPAD relative to the actual photon absorption time. The timing resolution of a SPAD (as well as an APD) is limited by both the avalanche build-up time and the transit time across the device [36,67]. The exponential current increase in Geiger-mode operation allows the SPAD to have the superior timing performance compared to the APD. Although the thin depletion region of a SPAD can hinder its PDE performance, especially as silicon technology nodes scale down, it offers the benefit of reduced transit time since the charge carriers will take less time to be collected at the edges of the depletion region. Also, with the increase of biasing voltage of the SPAD, the turn-on transient of the avalanche current becomes faster, typically lasting tens of picoseconds [68]. A timing jitter in the range of 10–100 ps can be achieved in a SPAD.

### 5.3. SPAD Design Considerations

#### 5.3.1. p-n Junction

**Figure 11:**
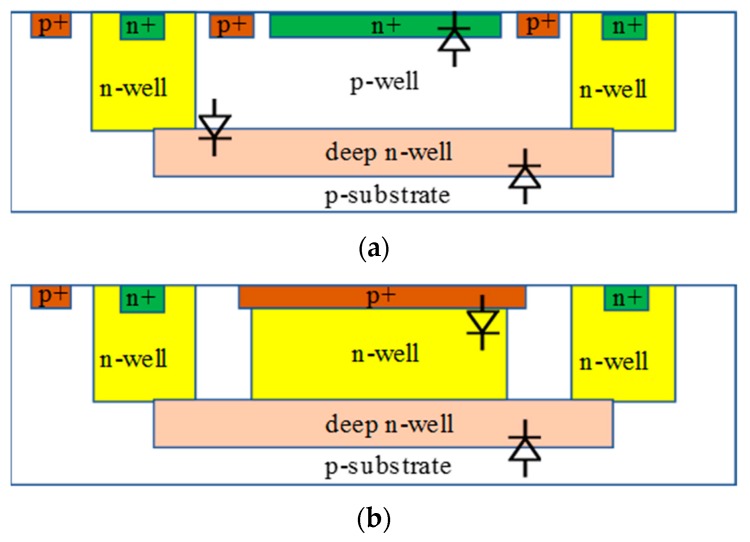
Cross-sectional view of p-n junctions in the standard TSMC 180 nm complementary metal-oxide (CMOS) technology: (**a**) Three diodes at the n+/p-well, p-well/deep n-well (DNW), and DNW/p-substrate junctions; (**b**) Two diodes at the p+/n-well and DNW/p-substrate junctions.

The most common scintillation crystal used in PET applications is L(Y)SO. This crystal converts the high energy gamma rays into visible light with a peak wavelength at 420 nm. In order to achieve the highest detection efficiency, the PDE peak of the SPAD should ideally be at the same wavelength. This peak wavelength is determined by the depth of the p-n junction depletion region since the absorption of certain wavelengths of light in silicon varies as a function of depth. However, doping concentrations and profiles are fixed in a given standard CMOS process by the foundry. In order to get the optimal PDE at a 420 nm wavelength for PET applications, different p-n junctions with varying depths in standard CMOS processes need to be investigated. Figure 11 shows the available options for p-n junctions in the standard TSMC (Taiwan Semiconductor Manufacturing Company) 180 nm CMOS technology, which include p+/n-well, n+/p-well, p-well/DNW, n-well/p-substrate and DNW/p-substrate. Comparing the PDE versus wavelength of different junctions, an optimal junction can be found at a given standard CMOS process.

In most SPAD applications, only one p-n junction is used for photon detection. However, multi-junction structures are also a possible solution for some applications [69,70,71]. The principle of this structure is that several p-n junctions are designed and stacked vertically, with all the junctions independently biased to ensure simultaneously operation. Compared to the one-junction structure, the extra junctions in this structure will increase the PDE. In [70], a dual-junction (p+/n-well and n-well/p-substrate) SPAD implemented in a 250 nm CMOS process achieved the highest PDE of 19% for the shallow diode at 400 nm and 29% for the deep diode at 500 nm. Another dual-junction (p-well/DNW and DNW/p-substrate) SPAD implemented in a 130 nm CMOS process achieved the highest PDE of 31% at 460 nm for the shallow junction, 30% at 660 nm for the deep junction and 38% at 640 nm when both SPADs were working [71]. Besides dual-junctions, the triple-junction SPAD is also an available option for standard CMOS technology. However, the multi-junction structures require complicated biasing circuits to ensure the multiple junctions work in the Geiger mode as well as an AC-coupled output strategy in order to feed the outputs of the SPADs into the following signal conditioning and processing circuits.

#### 5.3.2. Guard Ring (GR)

There usually exists a higher chance for early avalanche breakdown at the sharp edges of the depletion region depending on its layout and shape, known as premature edge breakdown (PEB). With PEB, the SPAD cannot be biased above breakdown voltage while maintaining a uniform electric field across its entire areal depletion region, which means the SPAD cannot function correctly. To overcome this issue, guard rings (GRs) have been employed in many SPAD designs. One important GR structure is shallow-trench isolation (STI).

In deep submicron (DSM) CMOS technologies, STI is traditionally used for providing the isolation between transistors and to also improve the density of transistors. In SPAD design, STI can be used to eliminate PEB in p+/n-well or p-well/n+ junctions since the edges of the depletion region are confined by the oxide trench to prevent the formation of sharp edges. The first STI-bounded SPAD implemented in a DSM non-high-voltage 180 nm CMOS technology was investigated in [72]. The silicon-dioxide trench guard ring structures was able to withstand a significantly higher electric field, resulting in reduced SPAD size, reduced space between pixels and thus improved FF. The electrical characterization of this STI-bounded SPAD showed a reliable operation over 5 × 10^10^ cycles at room temperature [72]. However, the STI is known to result in many defects at the boundary of SiO_2_-Si, which function as carrier generation-recombination centers. Therefore, if the STI directly contacts with the active region of the SPAD, the carriers generated at the interface can easily enter the depletion region of the diode, which results in an increase of the DCR and AP. In order to mitigate this issue, the authors in [73] showed a guard ring structure which combined the STI and a special p-well passivation in a 130 nm CMOS imaging sensor (CIS) process. Compared to the traditional STI-bounded SPAD, the DCR of the SPAD with this new STI guard ring was reduced by more than an order of magnitude. However, the feature of the p-well passivation implant is only available in a CIS process, which is more expensive than a standard CMOS process. In addition, masks (for example, the poly gate layer) stopping the formation of STI near the active region was also proved to be an effective method to alleviate this issue [57,74,75,76,77].

A diffusion GR structure is another structure widely used in SPAD design, such as the p-well diffusion GR for the p+/n-well junction and the n-well diffusion GR for the n+/p-well junction. The first SPAD with diffusion p-well implants as a GR was implemented in a high-voltage (HV) CMOS technology. The p+ implantation was enclosed by the p-well implants in an n-well. Due to the low doping profile in the diffusion p-well, the electric field at the device edges was reduced to ensure the initiation of the avalanche within the planar depletion region only. The same technique was also employed in SPAD designs in DSM standard CMOS processes [78,79,80]. A diffusion n-well guard ring structure was showed in [48]. The n+ implantation was surrounded by the n-well in a p-well. In order to reduce side effects of the STI, the n+ implantation extended into the n-well by 0.75 μm, resulting in the STI being positioned at a far enough distance away from the active region. In this way, carriers generated at the SiO_2_–Si interface are more likely to undergo recombination rather than diffuse into the active region, thus reducing the DCR.

A novel virtual GR in the 130 nm CMOS process was introduced in [57]. The virtual guard ring was implemented by using the retrograde doping profile of the deep buried n-well, which is an available feature in the triple-well CMOS technology (<250 nm). Due to the lower doping profile of the DNW to the surface, PEB can be prevented at the periphery of the p-n junction. Compared to a SPAD with a diffusion guard ring, the implementation of the virtual guard ring allowed the active area of the SPAD to be scaled down to a much smaller size [60]. With the use of a virtual guard ring, the SPAD size can be reduced to 2 μm with improved DCR, timing jitter and yield in the DSM technology because of the lower probability of having defects in a smaller active region [57].

#### 5.3.3. Quench and Reset Circuit

For proper operation, SPADs require a quenching circuit to stop the avalanche process (triggered either by a photon or noise), and a reset circuit to recharge the SPAD and bring it back to Geiger mode for the next photon detection. The time between the start and stop of the avalanche is called the “quenching time”. The reset time is the time needed to bring the SPAD back to the initial detection state by recharging the biasing voltage back to *V_EX_* + *V_BR_*. In general, the quench and reset circuits can be divided into two categories: passive quench and reset (PQR) and active quench and reset (AQR).

**Figure 12:**
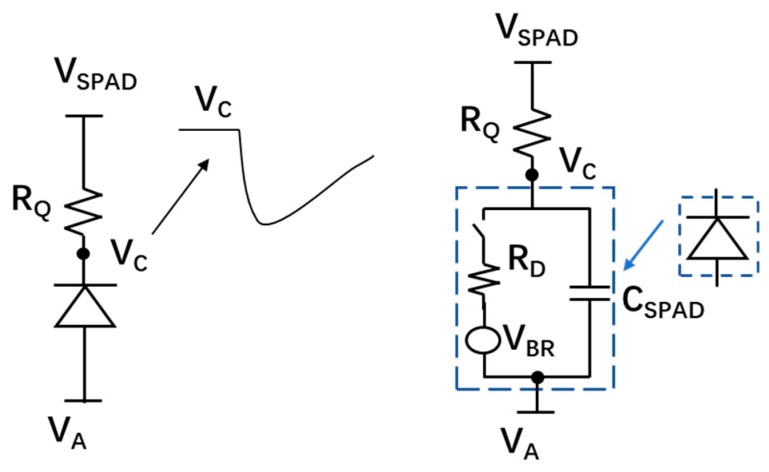
Simplified schematic for passive quench and reset (PQR) and the equivalent circuit simulation model.

The PQR circuits are generally implemented by using a large quench resistor. Figure 12 shows a simplified schematic of PQR and its equivalent model. As shown in the model, when avalanche occurs, the charges on the C_SPAD_ (capacitor of the SPAD) will discharge through the parallelly connected R_D_ (SPAD resistance) and R_Q_ (quenching resistor), thus resulting in an exponential decrease of the excess voltage with a final current approximately dependent on the bias voltage and the quench resistance. If this current is large enough, the avalanche is self-sustaining since enough charges are present in the depletion region, but below a certain value there exists a high probability that no carriers remain in the region after a random time, resulting in a quenched avalanche. Even though the value of this threshold is not well determined, a value of 100 μA is usually used in many SPAD analytic calculations and simulation models [49,81,82,83]. The threshold of the quenching current determines the minimum value for the quenching resistor, which usually ranges from 50 to 500 kΩ for the thin junction SPAD [84]. An approach by using the I-V characteristics measurement curve from the SPAD to determine the optimum value for the quench resistor was showed in [85]. PQR circuits have been widely used in SPAD pixel design since they are simple and only occupy a small area, resulting in a higher FF and decreased parasitics [86]. The PQR circuits are even employed in commercially available analog SiPMs. However, PQR does have some disadvantages. 

In PQR, the value of the quenching resistor should be large enough to ensure a fully quenched avalanche process, thus leading to a long recharge RC time constant. This time constant means a long dead time (DT) and reduced count rate for the SPAD. A typical value of DT for PQR is several hundred nanoseconds. For example, a SPAD with PQR circuits implemented in 130 nm CMOS process had a DT of 450 ns [87]. In addition, a long reset time can give rise to an early triggered avalanche during the voltage reset process, resulting in an undetectable low output or paralyzed DT behavior [88]. SPADs with a long reset time cannot function properly at a high count rate since the excess voltage is unable to recover to the originally biased value before the arrival of the next photon. In order to overcome these drawbacks, the AQR circuits have been intensively investigated in CMOS SPADs.

**Figure 13:**
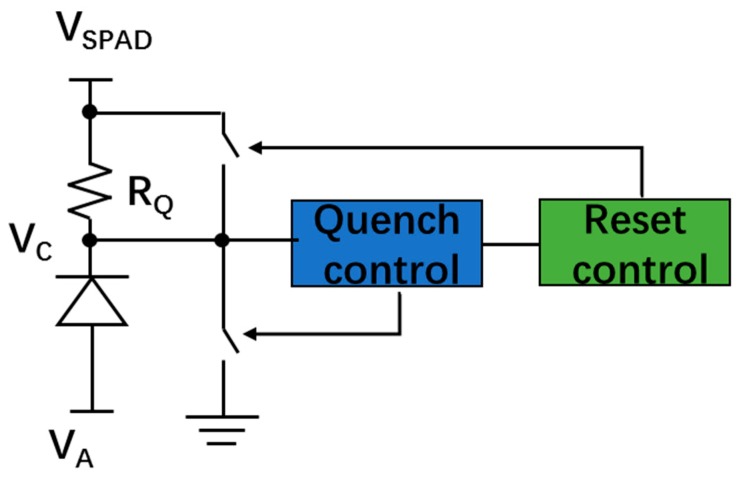
Simplified schematic for an active quench-reset (AQR) circuit.

AQR circuits have been widely employed to achieve a better performance in terms of DT [88], AP [54,89,90] and time-gating capabilities [54,91]. Figure 13 shows the basic concept of the active quench. When the avalanche is detected, the output of the SPAD will be used to generate the quench pulse to reduce the biasing voltage of the SPAD below the breakdown voltage, and then the reset pulse to bring the excess voltage back to the initial state. Generally, after quenching, the biasing voltage will stay below breakdown voltage for a predetermined time (hold-off time) before recharging. The carriers released from the trap centers within the hold-off time will not cause secondary pulses since the biasing voltage is below breakdown. A longer hold-off time leads to a reduced probability of AP, but also a longer DT, thus reducing the count rate of the SPAD. A typical value of the hold-off time for AQR is tens of nanosecond, which is generally much lower than the DT of PQR. The hold-off time of a SPAD with fully integrated AQR circuits (implemented in a HV 180 nm process) could be set as low as 10 ns [92]. Another drawback of AQR circuits is that they require some area to be implemented and are usually placed inside the pixel, ultimately reducing the pixel’s fill factor (FF). SPADs with AQR fabricated in early sub-micron CMOS processes usually had very small FFs due to the large size of transistors and design rule constraints. However, this limitation has been alleviated with the dimension shrinking of CMOS technology. A trend that FF increased with the scaling down of technologies was clearly shown in [93]. The FF is around 1% in 800 nm CMOS technology, 9% in 350 nm CMOS technology, 25% in 130 nm CMOS technology and 35% in 65 nm CMOS technology. However, compared to the advanced CMOS processes (e.g., 45 nm CMOS or 65 nm CMOS), SPAD pixel design prefers the less scaled technologies such as 180 nm CMOS and 350 nm CMOS, which have lower doping concentrations resulting in a thicker depletion region. As a result, higher photon detection probability (PDP) and a lower DCR can be expected. In order to deal with this dilemma, SPADs with a 3D structure have been proposed, in which the top tier of the semiconductor only has the SPAD array with improved photodetecting capabilities while the quenching and reset circuits can be implemented in the bottom tier usually with a smaller technology to benefit from their increased speed and lower power. Signals between the SPAD array on the top tier and the circuits on the second tier are connected by through-silicon vias (TSVs). A back-illuminated 3D stacked SPAD was reported in [93]. The SPAD arrays, implemented on the top tier by using 45 nm CIS technology, were stacked on top of a bottom tier containing the quench and reset circuits implemented in 65 nm standard CMOS technology. This design achieved a high FF up to 60.5% even though the SPAD was designed with conservative parameters. According to the achieved results, a future iteration of the SPAD can achieve an FF higher than 70% with optimized design parameters. However, the fabrication, assembly and packing are more costly for the 3D SPAD structure.

Table 3 lists the specifications of SPADs in the recent publications. From this table, we can see that the SPADs are implemented using various junctions (p-well/DNW, p+/n-well, p+/DNW, p-well/Niso, etc.) in different scaled CMOS technologies. Most the shapes of the active area of SPADs are circular in order to prevent PEB and the diameters of the active area are between 8 μm to 20 μm to ensure a reasonable DCR. The breakdown voltage is between 15 V and 30 V, which means that the biasing voltage for SPADs implemented in CMOS technologies is very easy to implement and safer when compared to the high operating voltage of PMTs. As the technology scales down, the breakdown voltage has a trend of decreasing due to higher doping profiles and thinner depletion of the junction. A PDE around 40% can be achieved with a moderate excess voltage at room temperature. The timing resolutions in CMOS SPADs are mostly less than 100 ps, which make SPADs favorable sensors for ToF PET applications.

### 5.4. Analog and Digital SiPMs

Compared with the single SPAD pixel design, the phenomenon of crosstalk needs to be considered in the applications of SiPM. Crosstalk happens when large SPAD pixel arrays with small pixel pitch are fabricated in one substrate to form a SiPM. When a Geiger pulse is triggered in a pixel cell, there is a finite probability that one or more neighboring pixels may also become triggered due to the electrical and/or optical crosstalk. Electrical crosstalk originates from avalanche-generated carriers diffusing laterally, entering the multiplication region of nearby SPADs. These carriers cause unwanted or spurious Geiger pulses. Electrical crosstalk can be reduced by dielectric and/or junction isolation. Optical crosstalk occurs from photons that are emitted during a Geiger pulse which can be reabsorbed in neighboring SPADs. The probability of crosstalk increases with increasing excess voltage. Crosstalk will result in the output signal being higher than that triggered by the amount of incident photons [94]. 

**Figure 14:**
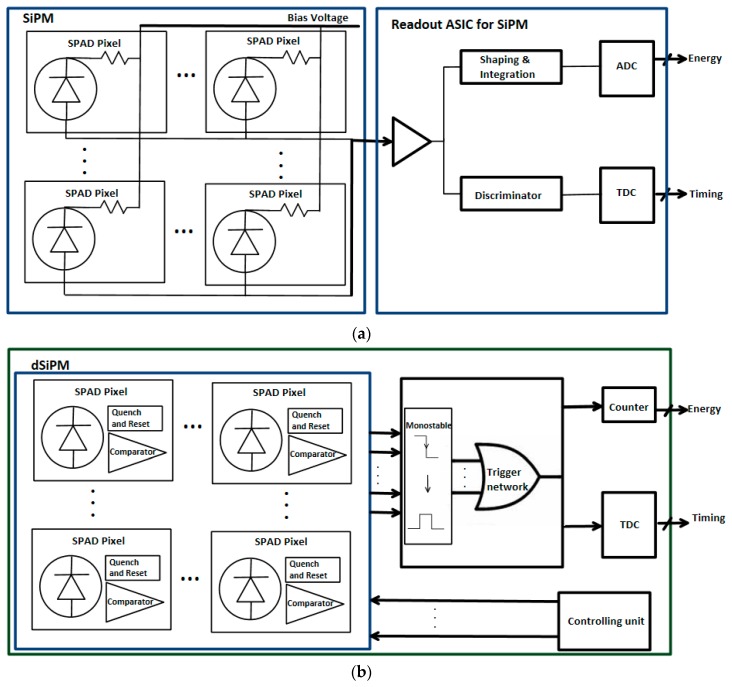
Readout electronics of a PET detector based on: (**a**) Analog SiPM and (**b**) Digital SiPM.

In the past decade, analog SiPMs was successfully employed in ToF PET systems [95,96,97,98,99]. In the application of PET, the detected signal from the analog SiPM needs a readout ASIC to get the timing and energy information of the detected photons, as shown in Figure 14a. The number of the output channels of analog SiPM can be reduced by employing resistive [100] or capacitive [101] multiplexing circuits. This feature reduces the efforts involved in designing the readout electronics for the analog SiPMs.

With progress in the design and fabrication of SPADs by using a standard CMOS process, the idea to integrate the SPAD array with the relevant circuits on one chip was proven and demonstrated by many researchers [102]. This new type of integrated sensor is called the digital silicon photomultiplier (dSiPM). In dSiPMs, not only are AQC circuits integrated with the SPAD array, but also other signal conditioning and processing circuits like digital acquisition circuits, time-to-digital converters (TDCs) and counters are also implemented on the same chip. Among these auxiliary circuits, the TDC is the most important one, greatly influencing the time resolution of the whole integrated chip. A detailed review of TDCs can be found in [103]. As shown in Figure 14b, the timing and energy information can be directly obtained from a single chip [104]. Due to the fully digital readout and processing circuits being integrated with the sensor on the same chip, the dSiPM offers excellent timing performance and eases the complexity of the signal conditioning and processing circuits which makes it an attractive sensor for medical imaging systems like PET. However, when dSiPM is compared to analog SiPM, there are a large number of electrical signals (i.e., clock, reset, digital data interfaces and some other auxiliary signals) that need to be connected when integrating the dSiPMs to form a sensor tile. Dealing with such a large number of connections can be challenging because of the limited number of pins of any ASIC or FPGA (field-programmable gate array) that is used. 

**Table 3:** Summary and comparison of single-photon avalanche diodes (SPADs) implemented by CMOS technology.

Reference, Year	Technology (nm)	Junction	V_BR_ (V)	Active Area (µm)	PDE (%)	Median DCR (Hz)	DT (ns)	After Pulse (%)	Time Resolution (ps)
[64], 2017	CIS 65 (BSI) ^a^	–	–	- (Square)	21.9 @ 660 nm Vex = 4.4 V	–	8	0.08 @ Vex = 4.4 V DT = 8 ns	95 @ Vex = 4.4 V (700 nm)
[105], 2012	CIS 90	p-well/DNW	14.9	6.4 (Circular)	44 @ 690 nm Vex = 3.5 V	~100 @ Low Excess Voltage	8	0.375 @ Vex = 0.36 V DT = 15 ns	82 @ Vex = 0.36 V (470 nm) 53 @ Vex = 1.36 V (470 nm) 51 @ Vex = 2.36 V (470 nm)
[60], 2009	CIS 130	p-well/DNW	14.4	8 (Circular)	28 @ 500 nm Vex = 1.4 V	25 @ Vex = 1.4 V 20 °C	–	–	–
[106], 2011	CMOS 150	p-well/Niso	23.1	10 (Circular)	31 @ 470 nm Vex = 5 V	~230 @ Vex = 5 V 25 °C	30	2.1 @ Vex = 5 V DT = 30 ns	170 @ Vex = 5 V (470 nm)
[106], 2011	CMOS 150	p+/n-well	16.1	10 (Circular)	26 @ 470 nm Vex = 3.5 V	~160 @ Vex = 3.5 V 25 °C	30	1.3 @ Vex = 3.5 V DT = 30 ns	60 @ Vex = 3.5 V (470 nm)
[65], 2017	CMOS 150	p+/n-well	18.01	10 (Square)	31 @ 450 nm Vex = 5 V	39 @ Vex = 3 V		0.85 @ Vex = 3 V DT = 150 ns	52 @ Vex = 4 V (468 nm) 42 @ Vex = 4 V (831 nm)
[61], 2018	CMOS HV 180	p+/shallow n-well	16.8	12.08 (Square)	55 @ 480 nm Vex = 4 V ^b^	28 @ Vex = 1 V 217 @ Vex = 4 V	–	–	260 @ Vex = 4 V (640 nm)
[107], 2010	CMOS HV 180	p+/DNW	20.3	8 (Octagonal)	20 @ 470 nm Vex = 3.5 V	180 @ Vex = 3.5 V	6	0 @ DT = 6 ns	80 @ Vex = 3.5 V (470 nm)
[108], 2014	CMOS 180	p-well/ DNW	23.5	12	>40 @ 440–620 nm Vex = 10 V ^b^	17 @ Vex = 2 V 1.45k @ Vex = 10 V	300	0.3 @ Vex = 10 V DT = 300 ns	70 @ Vex = 10 V (405 nm) 86 @ Vex = 10 V (637 nm)
[109], 2015	CMOS 180	p+/n-well/DNW	14.64	12 (Circular)	>40 @ 440–580 nm Vex = 4 V ^b^	31 @ Vex = 1 V 1.8k @ Vex = 4 V 25 °C	300	0.2 @ Vex = 4 V DT = 300 ns	95 @ Vex = 4 V (405 nm) 141 @ Vex = 4 V (637 nm)
[110], 2016	CMOS 180	p-well- epi-BN (Shallow PW)	36.5	–	27.8 @ 490 nm Vex = 4 V ^b^	2k @ Vex = 4 V 25 °C ^c^	–	0.34 @ Vex = 4 V DT = 300 ns	427 @ Vex = 2 V (637 nm) 223 @ Vex = 4 V (637 nm) 243 @ Vex = 2 V (405 nm) 141 @ Vex = 4 V (405 nm)
[110], 2016	CMOS 180	p-well- epi-BN	25.46	–	>40 @ 460–600 nm Vex = 11 V ^b^	40 @ Vex = 4 V 25 °C ^c^	–	7.2 @ Vex = 11 V DT = 300 ns	139.5 @ Vex = 3 V (637 nm) 100.8 @ Vex = 11 V (637 nm) 133 @ Vex = 3 V (405 nm) 97.2 @ Vex = 11 V (405 nm)
[111], 2013	CMOS 350	–	25	20	28 @ 570 nm Vex = 6 V ^d^	25 @ Vex = 6 V Room Temp	20	1.3 @ Vex = 6 V DT = 20 ns	–
[112], 2009	CMOS 350	p+/DNW	27.5–28.4	–	–	4k @ Vex = 4 V Room Temp ^e^	–	4.5 @ Vex = 5 V DT = 500 ns	–

^a^ The SPAD is implemented using a 3D structure. The SPAD is using back-side illumination CMOS imaging 65 nm technology and the circuits are implemented in standard CMOS 40 nm technology. ^b^ The values are the PDP (not PDE). ^c^ The values are read from the curves. ^d^ The highest PDE is 55%, but the measurement setup is not revealed in the publication. ^e^ 60% of the 17 samples had a DCR lower than 4 kHz with an excess voltage of 4 V at room temperature.

### 5.5. PET Applications

#### 5.5.1. SiPMs

In this section, SiPMs refer only to analog SiPM. Digital SiPMs (dSiPMs) will be discussed in the next section (Section 5.5.2). SiPMs were successfully employed in some of the latest commercial PET/CT and PET/MRI systems. In the Discovery MI PET/CT system (the latest PET/CT system from GE), a 2 × 3 array of SiPM pixels (4 × 6 mm^2^) are assembled to form a large active area SiPM device (12.6 × 12.6 mm^2^), with a custom ASIC designed to process the output signals. The same sensor technology was also used in the latest SIGNA™ PET/MRI from GE. Currently, the most advanced PET system in the commercial market is the Biograph Vision PET/CT system from Siemens, in which SiPMs are chosen for the PET detector (more details about the latest PET/CT and PET/MRI systems are provided in Section 7). In parallel to the commercial success of SiPM-based systems, research is also being carried out such as in two EU granted projects to build PET inserts for human brain imaging where SiPMs were chosen as the photodetectors.

One project called the Multimodal Imaging of Neurological Disorders (MINDview) developed a brain PET system with high sensitivity and resolution to visualize the pathways of neurotransmitters and the disruptions of the pathways associated with mental disorders. The PET scanner was required to be compact and insensitive to magnetic fields in order to function as a PET insert to most of the existing MRI systems to achieve hybrid PET/MRI systems. The MINDview PET scanner was made of three stacked detector rings, with each ring having 20 detectors. The complete system was designed to achieve a FOV of ~15 cm in the axial direction and a transaxial diameter of ~33 cm. In order to determine the configuration of the PET detector for the MINDview PET system, two types of detector designs were compared in [113] to study the feasibility of SiPM arrays. The scintillator in the first type of the detector was a three staggered-layer pixelated LYSO array with the configuration of a 35 × 35 array, 36 × 36 array, and 37 × 37 array from the top to the bottom. The size for each single scintillator was 1.5 × 1.5 × 6.0 mm^3^ and MicroFB-30035-SMT (SensL) SiPMs were used. The outer dimension of these SiPMs was 4 × 4 mm^2^ while the active area was 3 × 3 mm^2^. The SiPMs were arranged to form a 12 × 12 array to cover an active area of 50.2 × 50.2 mm^2^ to decode the pixelated LYSO array. Another type of the detector was made of a monolithic LYSO crystal with a size of 50 × 50 × 20 mm^3^ and a 12 × 12 array of MicroFC-30035-SMT (SensL) SiPMs. The output analog signals from the SiPMs were processed through a charge division method and a customized analog-to-digital converter (ADC) board. By using a ^22^Na point source, the detector with pixelated scintillators had an energy resolution of 11.8% FWHM for the top layer, 9.6% FWHM for the middle layer and 10.2% for the bottom layer at 511 keV. The DOI resolution was viewed to be equal to the thickness of the layers (6 mm) since the flood map of the detector showed that the three layers of the scintillators were clearly resolved. The experimental results showed that the detector with the pixelated scintillator block was capable of acquiring PET images with 1 mm resolution in the center of the brain. The detector with the monolithic crystal had an averaged detector spatial resolution of 1.5 mm, a DOI resolution of <2 mm and an averaged energy resolution of 17% when all faces except the one coupled onto the SiPMs were painted back. The PET detector with the monolithic crystal block was chosen for the final system integration due to its capability to offer adequate intrinsic resolution at a more affordable cost. The preliminary performance of the integrated MINDview PET insert was measured and evaluated inside a 3T MRI system (Siemens mMR) [114]. The PET scanner was defined to have an effective FOV of 240 mm diameter in the transaxial direction and 154 mm along the axial direction. In the integrated system, the SiPMs was customized into a MINDView-series type (similar to J-series from SensL). Similarly, the SiPMs with 3 × 3 mm^2^ active area were placed in a 12 × 12 array with a pitch of 4.36 mm to couple with a monolithic LYSO crystal of size 50 × 50 × 20 mm^3^. By using a phantom with FDG filled in, an average energy resolution was measured to be 17.5 ± 1.5% for the entire system. The system sensitivity was ~7% within an energy window of 350–650 keV at the center of the FOV by moving the 1 mm ^22^Na point source along the axial direction with a 0.5 mm step. The DOI resolution was expected to be 4 ± 1 mm for all 60 detectors. In order to measure the spatial resolution, a small ^22^Na point source (0.25 mm diameter) was placed along the radial direction at three positions along the axial direction, the center of FOV (CFOV), and 0.25 and 0.375 of the axial axis. The spatial resolution at the CFOV was measured to be ~1.7 mm along the three directions, but the spatial resolution at 100 mm-off the radial distance degraded to 3 mm in the axial and radial directions and 2.2 mm in the transversal direction. The magnetic compatibility measurements showed that 2.5 mm rods of a Derenzo phantom could be clearly resolved and there was no degradation of count rate under a variety of MRI acquisition sequences.

In another project named TRIMAGE, a trimodal PET/MRI/electroencephalogram (EEG) integrated system is being developed. The combination of PET for highly sensitive molecular imaging, EEG for temporal information, and functional MRI to reveal brain activity and provide high-resolution anatomical structural images made the TRIMAGE system a powerful tool for the diagnosis and treatment of mental disorders [115]. The PET ring of the TRIMAGE system was made of 18 PET detectors, each with an active area of 54 × 162 mm^2^. Each detector module consisted of four tiles, and each tiles was made of a two-layer half-pitch staggered LYSO scintillator (7 × 7 LYSO array with a size of 3.3 × 3.3 × 8.0 mm^3^ on the top layer and 8 × 8 LYSO array with a size of 3.3 × 3.3 × 12.0 mm^3^ on the bottom layer) and two arrays of NUV-SiPMs (AdvanSiD) [116]. Each array was an 8 × 4 matrix of the SiPM cell with an active area of 3 × 3 mm^2^ and an outside dimension of 3.4 × 3.4 mm^2^. The 64 SiPM outputs from one tile were processed by a 64-channel TRIROC ASIC [117]. The preliminary measurement showed that the PET detector could provide a CRT of 420 ps FWHM and an energy resolution of 10.7% with a single crystal (3.3 × 3.3 × 8.0 mm^3^) setup. As for the two-layer staggered scintillator block setup, the detector had an average energy resolution of 18% for the crystals on the bottom layer and 16% for the crystals on the top layer [118]. Moreover, the flood map of the two-layer staggered scintillator block showed that all crystals were clearly resolved. The detector performance with the final version of the readout and data acquisition electronics showed an energy resolution of 22% (top layer) and 20% (bottom layer). By using an energy window of 350–650 keV and a coincidence window of 3 ns, the measured CRT for the two tiles of detectors was 529 ps (top layer) and 501 ps (bottom layer) when the SiPMs were biased at an optimal voltage in [119]. The TRIMAGE research team are currently working on the system integration and validation.

The total-body PET scanner EXPLORER is another interesting project based on SiPM technology, to develop a PET scanner with an axial length long enough for a total human body scan at one time [120,121]. The EXPLORER PET system showed an effective sensitivity 40-fold higher than that of the current commercial PET scanner, giving it the capability to obtain a PET image in a short time (< 1 min) and perform the PET scan with a very low dose of the radiotracer. The system was designed to have a total axial FOV of 194 cm with a 76 cm diameter bore. There were 8 PET rings for the entire system. Each ring consisted of 24 detector modules and each module contained 5 × 14 detector blocks. A 6 × 7 array of LYSO scintillators with a size of 2.76 × 2.76 × 18.10 mm^3^ was coupled on a 2 × 2 array of SiPMs (6 × 6 mm^2^, SensL J-series). In total, the system contained 53,760 SiPMs and 564,480 LYSO scintillators. The detectors achieved an average energy resolution of 11 ± 1.5% and a CRT of 409 ± 39 ps [122]. The prototype of the total-body PET scanner was integrated into a commercial PET/CT system (uEXPLORER PET/CT) [123] and granted FDA approval [124]. 

SiPMs are also being used in many small-animal PET systems. A small-animal PET based on monolithic LYSO scintillators was introduced in [125]. The system consisted of eight detector modules and each module was made of a monolithic LYSO crystal with a pyramidal shape (48 × 48 mm^3^ entrance surface area, 50 × 50 mm^3^ exit surface area, 10 mm thickness) and a 12 × 12 array of SiPMs (SensL C-Series type). By using a spherical ^22^Na with the diameter of 0.25 mm, the system had a measured DOI resolution of 2 mm FWHM for all eight detectors, a spatial resolution of ~1 mm FWHM in the entire FOV range, and a sensitivity of 2.8% with the energy window between 358 and 654 keV. The average energy resolution was ~15% at 511 keV at 22℃ and ~14% at 15℃. 

In Table 4, a summary of SiPMs for PET applications in recent publications is given. For the application of SiPMs, ASICs are usually used to process the analog output of SiPMs to get the energy and timing information about the interaction of gamma rays with the scintillator. Many readout ASICs for SiPMs were also introduced in recent publications such as Triroc 1A (Weeroc) [117], Petiroc 2A (Weeroc) [126], PETsys TOFPET2 ASIC [127], and the Position Energy and Timing ASIC (PETA) series [128,129,130,131]. A more detailed summary of the applications of ASICs for SiPMs can be found in [132].

#### 5.5.2. dSiPMs

The Digital Photon Counter (DPC) is a commercialized architecture developed by the Philips Digital Photon Counting (PDPC) team. The dSiPM of the DPC3200-22 is currently the only fully digital integrated SiPM and has already been successfully employed in PET imaging systems developed by Philips. This sensor chip contains a 2 × 2 pixel array. Each pixel is comprised of four sub-pixels, each containing a 32 × 25 array of SPAD cells, totaling 3200 cells. The triggers from the four subpixels due to the first photon detection or the first dark noise detection are logically combined to produce one master trigger to the main controller and the TDC. By changing the logical combinations of four triggers from each subpixel, the master trigger can be generated through four different trigger schemes. These trigger schemes are:

- Trigger scheme 1 = ST_1_ ∨ ST_2_ ∨ ST_3_ ∨ ST_4_,
- Trigger scheme 2 = [(ST_1_ ∨ ST_2_) ∧ (ST_3_ ∨ ST_4_)] ∨ [(ST_1_ ∨ ST_4_) ∧ (ST_2_ ∨ ST_3_)],
- Trigger scheme 3 = (ST_1_ ∨ ST_2_) ∧ (ST_3_ ∨ ST_4_),
- Trigger scheme 4 = ST_1_ ∧ ST_2_ ∧ ST_3_ ∧ ST_4_,

where ST_1_, ST_2_, ST_3_, ST4 are the triggers from four subpixels. The details of logical interconnections between the triggers from the four subpixels can be found in [133]. The four pixels share 2 TDCs and one main acquisition controller. The master trigger is used for the start signal of the TDC while the stop signal for the TDC is from the reference clock. In order to avoid losing data because of the metastability status (when the start and stop signal encounter a coincidence), the two TDCs run with complementary clocks. By using this configuration, at least one valid timestamp is recorded. The FF of the chip is 82.9% for the SPAD array and 77.7% for the whole system. It achieves a PDE >30% at the 420 nm wavelength and the timing resolution for the TDC was 23.5 ps with a dynamic range more than 11 ns [134]. 

The die of the DPC-3200-22 can be arranged on a tile to form an array for practical use. Taking the DPC3200-22-44 as an example, the tile consists of an array of 4 × 4 dies (DPC-3200-22). The relevant signal conditioning and processing printed circuit board (PCB) is placed beneath the tile. Measurements and prototypes of PET based on the DPC series have been widely investigated by many research groups in recent years [135,136,137,138,139,140,141,142,143,144].

A PET prototype by using the DPC-3200-22-44 was showed in [135]. Measured with a ^22^Na point source, the prototype demonstrated an excellent CRT of 266 ps FWHM within a 440–660 keV energy window and an energy resolution of 10.7% FWHM. Even with the large size (4 × 4 × 22 mm^3^) of LYSO crystals, the system showed a spatial resolution close to 2.4 mm FWHM. By using a small sized (3 × 3 × 5 mm^3^) LSO:Ce,Ca crystal, the detector showed a CRT as low as 120 ps FWHM, which was extremely low [145]. A later version of the DPC (DPC-6400-44-22) was measured to have a spatial resolution below 1 mm in the center by using a 24 × 24 × 10 mm^3^ LSO:Ce,Ca crystal [136]. In 2015, the feasibility to combine two types of scintillators - LYSO and GAGG (Gadolinium Aluminum Gallium Garnet) with the DPCs (DPC-3200-22-44) for PET application was investigated in [137]. By coupling individual LYSO scintillators of 2 × 2 × 6 mm^3^ onto each pixel of the DPC, the CRT was 171 ps FWHM within an energy window of full-width-at-tenth-maximum (FWTM) of the 511 keV peak, and the energy resolution was 12.6% FWHM. Using same sized GAGG scintillators in this set-up, the CRT was 310 ps FWHM (511 keV peak FWTM energy window) and the energy resolution was 8.5% FWHM. Two PET detectors were made by equipping the GAGG array (3.2 × 3.8775 × 8 mm^3^) on the DPC of the same size. In order to emulate a detection ring of 10 modules, the testing phantoms were placed on a rotatory platform in the field of view. The CRT of the PET system was 619 ps FWHM (511 keV peak FWTM energy window) and the energy resolutions was 9.2% FWHM. Even though the CRT using GAGG was much lower than that using LYSO, the system achieved a relative low energy resolution. That is to say, the detector made of the DPC and a GAGG scintillator was an alternative option for small animal PET with a small FoV, in which the ToF technique—which requires high performance in terms of CRT—is not a critical issue for image resolution. Another PET prototype with a FOV of 18 cm diameter and an axial height of 20 cm, formed by 12 detector modules, was introduced in [138,139]. Each module comprised of an array of 16 × 16 LYSO of size 1.85 × 1.85 × 10 mm^3^ and a DPC-3200-22-44. The PET system was measured to have a CRT of 298 ps and a spatial resolution of 1.6 mm FWHM by placing a hot rod phantom in the CFOV [138,139].

DPC dSiPMs were also used in the HYPERION II**^D^** PET/MRI insert. In this system, the DPC-3200-22 sensors were arranged to be a 4 × 4 array with self-designed readout electronics to form a sensor tile. In [146], two types of scintillators were measured. The preclinical type of detector was made of a 30 × 30 array of LYSO scintillators (1 mm pitch, 12 mm height) coupled onto the DPC array through a light guide (2 mm thick). The clinical type of detector consisted of an 8 × 8 array of LYSO scintillators (4 mm pitch, 10 mm height) and a 4 × 4 array of DPCs. By using point-like ^22^Na sources (0.25 mm diameter), trigger scheme 1 of the DPC and a narrow energy window (500–520 keV), the system with the preclinical configuration showed a CRT of 240.4 ps FWHM and the system with the clinical configuration achieved a CRT of 208.4 ps FWHM with the use of correction methods for crystal delay and time walk. With the configuration of trigger scheme 2 and an energy window of 250–625 keV, the CRT was 436.1 ps for the preclinical type and 289.4 ps for the clinical type. By using point ^22^Na sources, the preclinical system achieved an average energy resolution of 12.6% FWHM, a CRT of 565 ps FWHM (trigger scheme 3 and an energy window of 411–561 keV) and an average volumetric spatial resolution of 0.73 mm^3^ in the CFOV [147]. The 0.8-mm rods in a hot-rod phantom were clearly resolved by using an MLEM reconstruction. The system also showed good compatibility with the MRI system. The performance evaluation of the preclinical system based on the National Electrical Manufacturers Association (NEMA) NU4-2008 standard was introduced in [148]. By moving a point ^22^Na source along the radial direction at the center and 0.25 (22.5 mm) of the axial axis with an energy window of 250–625 keV and a 2 ns coincidence time window, the system achieved a spatial resolution of 1.7 mm FWHM nearby the isocenter and 2.5 mm FWHM at 50 mm off the center with the FBP reconstruction method, an averaged energy resolution of 12.7% FWHM at 511 keV and CRT of 609 ps FWHM (using trigger scheme 3).

**Figure 15:**
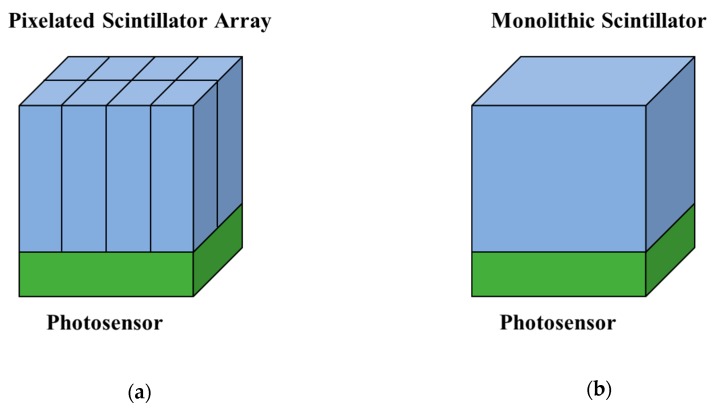
Illustration of a positron emission tomography (PET) detector: (**a**) pixelated scintillator (2 × 4 array) mounted on a photosensor; (**b**) monolithic scintillator mounted on a photosensor.

The DPC can also be combined with a monolithic scintillator for promising results in PET applications. Compared to the finely pixelated array, less efforts are usually required to integrate the monolithic crystals into the PET detectors. As shown in Figure 15, the monolithic crystals provide higher sensitivity because there is no inter-pixel dead space which is unavoidable in the pixelated design. Moreover, the DOI information can be derived from the light distribution shape across the monolithic crystals without modifying the crystals. This information leads to more accurate LORs, eventually resulting in a higher spatial resolution for PET systems. The first preclinical PET system based on thin monolithic scintillators was proposed in [140]. The system was designed for rat-brain imaging and thus had a very compact size. Four detectors were arranged as a square and the distance between two opposing modules was 34.5 mm. The field of view of the system was 32 × 32 × 32 mm^3^. Each detector comprised of a thin monolithic LYSO scintillator (32 × 32 × 2 mm^3^) optically coupled onto a DPC-3200-22-44. The whole system achieved a CRT of 680 ps FWHM by using an energy window of 400–650 keV, an energy resolution of 18% FWHM and a spatial resolution of 0.7 mm FWHM at the center by moving a calibrated point ^22^Na sources (0.25 mm diameter) along the radial axis in the center of the axial FOV (from 0 mm to 10 mm with a step of 1 mm). In order to improve the spatial resolution, a thicker monolithic crystal was coupled to the DPC-3200-22-44. After applying a mean nearest neighbor positioning algorithm and a DOI decoding algorithm, the detector showed an intrinsic spatial resolution of 0.6 mm FWHM, an energy resolution of 23% FWHM and CRT of 529 ps FWHM with the same energy window of 400–650 keV. The DOI resolution was 1.66 mm FWHM [144].

A supervised machine learning algorithm based on gradient tree boosting (GTB) was also developed in [149] to enhance the detection of the position of interaction of the gamma ray with the monolithic scintillator. The GTB model was trained by the data from the measurement of a monolithic LYSO with a size of 32 × 32 × 12 mm^3^ and achieved a spatial resolution of 1.40 mm in the *x*-direction and 1.24 mm in the *y*-direction (corrected for finite beam size). A DOI estimation method based on GTB was described in [150]. With a 32 × 32 × 12 mm^3^ monolithic scintillator, the GTB model showed a uniform spatial resolution of 2.12 mm FWHM and an averaged mean absolute positioning error of 1.28 mm along the total 12 mm depth of the scintillator.

The applications of DPC-3200-22-44 coupled with a thick monolithic LYSO:Ce scintillator of size 32 × 32 × 22 mm^3^ was reported in [141]. The first detector prototype they reported was a dual-sided readout (DSR) configuration, in which two DPCs (DPC-3200-22-44) were coupled onto the 32 × 32 mm^2^ front and back faces of the crystal, respectively. The DSR detector was measured to have a CRT of 147 ps FWHM (511 keV peak FWTM energy window), a spatial resolution of 1.1 mm FWHM and an energy resolution of 10.2% FWHM. The DOI resolution was around 2.4 mm. They also reported a conventional prototype of the detector which consisted of a DPC-3200-22-44 coupled with one thick monolithic LYSO:Ce scintillator (32 × 32 × 22 mm^3^). The detector resulted in a CRT of 214 ps FWHM (511 keV peak FWTM energy window), an energy resolution of around 10.2% FWHM and a spatial resolution of 1.7 mm at the center [142]. The spatial resolution in the system level was ~2.9 mm at the center of the FoV by employing a 2D FBP method. By using the DOI information, the system had an almost uniform distribution of spatial resolution from the center of the FoV up to a radial distance of 25 cm, where the radial spatial resolution was measured to be ~3.3 mm FWHM and the tangential spatial resolution was ~4.7 mm FWHM [143]. Table 5 shows a summary of the application of the DPC series to different types of PET detectors and Table 6 summarizes some applications of DPC dSiPMs for PET systems. The excellent performance of the DPC series in CRT, energy resolution and sub-millimeter spatial resolution make them excellent dSiPMs for PET applications.

Besides the commercially available DPC series, many other types of dSiPMs have also been designed and investigated for PET applications among different research groups [19,151,152,153,154,155]. The dSiPM from [80] was composed of 2 × 2 identical subpixels, each with an array of 64 × 32 SPADs. The size for each SPAD was 52 × 30 μm^2^ including the electronics. The FF for each cell was 50%. A peak PDE of 31% was achieved at 420 nm with an excess voltage of 3.3 V. The averaged DCR for a single cell was reported to be as low as 150 Hz and the total DCR of the sensor was around 200 kHz at 20 °C. The start signal for the on-chip TDC was from the first photon detected by the subpixels. The stop signal was the system clock, which functioned as a time reference for all TDCs. Two dSiPMs coupled with a 3 × 3 × 5 mm^3^ LYSO scintillator were set up for the PET coincidence measurement. By using a ^22^Na point source, the setup achieved an energy resolution of 10.7% FWHM at 511 keV and a CRT of 153 ps FWHM.

A 14 × 10 array of mini-SiPMs integrated with TDCs implemented in 0.35 µm high-voltage (HV) CMOS technology was reported in [78]. Each mini-SiPM comprised of 32 SPAD cells, which contained the SPAD itself, controlling circuits (used to disable pixels with high DCR), a passive quench NMOS, several switches and a standard 6-transistor (6T) SRAM. The 6T SRAM can be configured to send a signal to the controlling circuits to disable the SPAD cell if the SPAD cell was found to be noisy due to fabrication impurities and defects. The FF for the SPAD cell itself was 39%, and 29% including the electronics. One 10-bit TDC based on a ring oscillator was shared by the SPADs in one column and was triggered by the first photon detected by the column. The simulations indicated a time resolution of 325 ps FWHM. 

A multichannel dSiPM comprised of a 16 × 26 SPAD array and an array of 48 TDCs using a 0.35 µm high voltage (HV) CMOS technology was reported [151]. This multichannel (column-parallel TDC) configuration enabled the acquisition of multiple timestamps for each gamma ray event, which exhibited more tolerance to DCR in ToF PET applications [156]. In this dSiPM, 3 TDCs were shared by 26 SPAD cells in one column. One cell contained a SPAD, an active reset circuit, a 2-bit counter, a masking circuit, and a memory, which occupied an area of 30 × 50 µm^2^ with a 21.2% FF. When biased with an excess voltage of 4 V, the peak PDE for the SPAD was around 30% at a 420–430 nm wavelength and while at 20°C, the median DCR was about 250.0 Hz/µm^2^. The TDC achieved a time resolution (LSB) of 51.8 ps, an averaged differential non-linearity (DNL) of 1.97 LSB, and an averaged integral non-linearity (INL) of 2.39 LSB after compensation. The system timing jitter was measured to be 264 ps FWHM by applying a 34 ps laser pulse with the wavelength of 405 nm.

In [19], a dSiPM was developed for PET applications in a 0.13 µm CMOS imaging technology. This sensor chip contained 8 × 16 array pixels, and each pixel was comprised of four mini-SiPMs. Each mini SiPM had 180 SPADs (12 × 15 array). In order to increase the pixel FF and reduce the number of TDCs, compression techniques were employed. In spatial compression, 3 SPAD cells were OR’ed together to generate only one output. After spatial compression, the number of outputs from the mini-SiPM were reduced from 180 to 60. The 60 outputs were processed by temporal compression, in which the output triggers were connected to a monostable circuit to reduce the width of the pulse to as short as 250 ps, then combined into one output by an OR tree. After compression, four mini-SiPMs shared only two TDCs. However, the compression techniques would inevitably result in some compression loss. The system evaluation had a total compression of 10% in a PET-like setup. This chip was able to detect the timestamp and energy of events at a rate of 100M samples per second. The SPAD was implemented in a circular shape and with an active diameter of 16.27 μm. The SPAD array achieved an FF of 42.6% in the SPAD array itself and 35.7% in the whole chip. The median DCR was 108.7 Hz/µm^2^ and the highest PDE was 45% with an excess voltage of 1.5 V. The 12 bits TDC achieved a resolution of 64.56 ps, and the DNL in a 50 ns range was less than 0.28 LSB. The ToF performance was measured by using a 3 × 3 × 5 mm^3^ LYSO at 20°C. The system achieved an energy resolution of 10.9% and a CRT of 399 ps FWHM.

Table 7 summarizes the dSiPMs in the recent publications. Not all dSiPMs in the table are specifically designed for PET. The dSiPMs reported in [152,153] were designed for fluorescence lifetime imaging microscopy (FLIM) and the dSiPM in [154] was for spacecraft navigation and landing (SNL). Even though these applications may function at different light wavelengths from that of PET applications, they also require dSiPMs to offer good performance in terms of high PDE, low DCR, good linearity, and low timing jitter, which are similar requirements for PET applications. Thus, the dSiPMs designed for other applications are still valuable references for the design of dSiPMs for PET applications.

## 6. Cadmium Zinc Telluride (CZT) Detectors

### 6.1. Operating Principles of CZT

**Figure 16:**
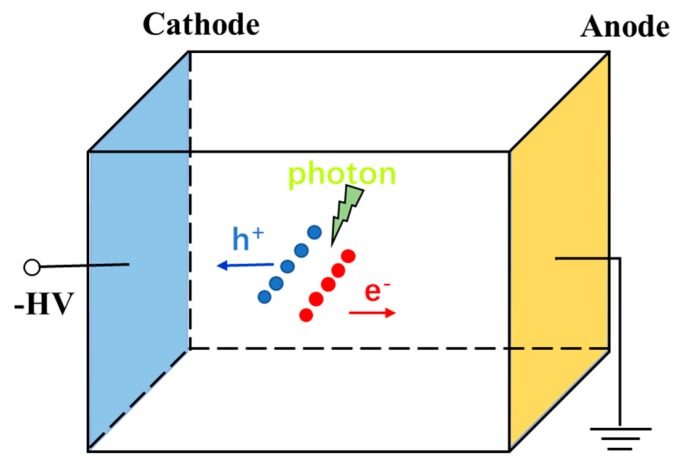
Planar illustration of a cadmium zinc telluride (CZT) detector. A negative high voltage is applied on the cathode and the anode is grounded. The e–h pairs are generated by absorbing the energy from the gamma rays.

Semiconductor detectors have gained attention in the application of nuclear radiation detection during the last few decades since they have the ability to convert high-energy rays such as x-rays and gamma rays directly into electronic signals. Unlike the scintillator, the direct conversion of the semiconductor detector can avoid the stochastic effects resulting from the light generation, propagation and conversion into electronic signals, which makes it a favorable alternative to the scintillator-based photon detector [157]. Among the semiconductor detectors, CZT detectors have shown great potential in PET imaging systems. Compared to the L(Y)SO scintillators, which have a mass attenuation coefficient of 0.117 cm^2^/g at 511 keV (Compton scattering mass attenuation coefficient of ~0.038 cm^2^/g and photoelectric absorption mass attenuation coefficient of ~0.073 cm^2^/g), the CZT detectors show slightly lower performance in the absorption of high-energy gamma rays. The mass attenuation coefficient of CZT detectors is 0.086 cm^2^/g at 511 keV. The fraction of Compton scattering is 0.82 and the fraction of photoelectric absorption is 0.18, which translates into a Compton scattering mass attenuation coefficient of ~0.071 cm^2^/g and a photoelectric absorption mass attenuation coefficient of ~0.015 cm^2^/g [158]. Despite this, the relatively high atomic number (average Z of 49.1 for Cd_0.9_Zn_0.1_T) allows a good portion of high energy gamma rays be absorbed to generate a large enough signal, which is distinguishable from the noise. The high material density (5.78 g/cm^3^) further yields high stopping power. Moreover, the wide band-gap (1.6 eV) allows the CZT detector to operate at room temperature [159,160,161]. Figure 16 shows the operating principles of a CZT detector. The interaction of the incident gamma ray with the crystal lattice of CZT produces primary electrons. The high-energy primary electrons will undergo impact ionization to generate secondary charges. In semiconductor detectors like CZT, the generated secondary charges are pairs of electrons and holes, with electrons excited from the valence band into the conduction band and the holes left in the valence band. The number of e-h pairs created by impact ionization is proportional to the deposited energy from the incident gamma ray and the energy needed to create an e-h pair in the semiconductor (the e-h pair creation energy for CZT is 4.64 eV). Due to the high electric field from the applied external voltage between the anode and cathode, the electrons and holes will drift and be collected by the anode and cathode, respectively, giving rise to the signal current. Then, the signal is processed by the readout electronics.

**Figure 17:**
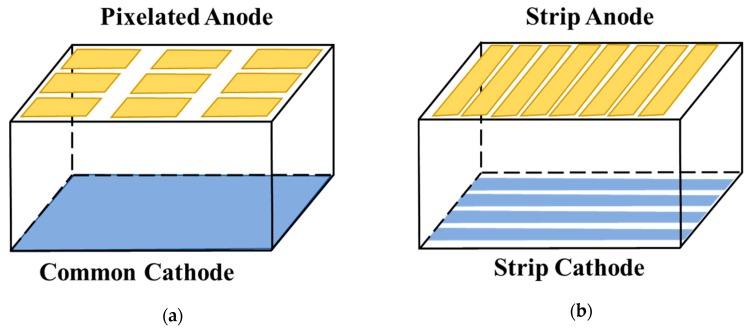
Illustration of electrode patterns of a cadmium zinc telluride (CZT) detector: (**a**) pixelated pattern; (**b**) cross-strip pattern.

In the CZT detector, both the electrons and holes will drift to the electrodes to generate current which represents energy information. However, the mean drift length of electrons is much larger than that of holes in the CZT detectors. This makes the anode the favorable collector for energy information. Two types of electrode patterns are commonly used in the CZT detector as shown in Figure 17. Figure 17a shows the pixelated pattern design, in which only the anode comprises of an array of small pixelated electrodes and the cathode is one shared large electrode. The outputs of the pixelated electrodes produce the position information of interaction. Figure 17b shows the cross-strip electrode pattern. In this configuration, the same 2D position information can be read from orthogonally crossed anode and cathode strips. Compared to the pixelated configuration, the cross-strip configuration will reduce the numbers of electrodes required to produce the same position resolution across the same volume of the detector (from N2 down to 2N), which greatly relieves the burden for the readout electronics [162].

### 6.2. CZT Detector for PET Applications

A large amount of research [163,164,165,166,167,168,169,170,171,172] has been conducted regarding the potential of CZT detectors for PET applications by investigating the electrode design, detector design, system integration and algorithm development to increase the energy resolution, improve the spatial resolution and reduce the coincident time.

#### 6.2.1. Pixelated Electrodes

The preliminary performance of a CZT-based PET system developed at the Korea Atomic Energy Research Institute (KAERI) was presented in [164]. The prototype PET system consisted of two pixelated CZT detectors and a discrete type data acquisition (DAQ) system. Each CZT detector had 64 (8 × 8) pixelated anodes (2.2 mm anode pixel pitch) and a common cathode with the volume size of 19.4 × 19.4 × 6.0 mm^3^. The DAQ system comprised of a charge-sensitive amplifier with a charge gain of 8 mV/fC and equivalent noise charge (ENC) of 55 electrons, a CR-RC^4^ shaping amplifier with a peak time of 5 μs, and an ADC driver. The DAQ system also had a 65 M samples/s flash ADC, a self and external trigger, and a USB 3.0 interface. In order to obtain the system performance, a gamma-ray point source was located at the center of the two CZT radiation detectors. After setting the energy window between 495 keV and 526 keV, an averaged energy resolution of 3.75% was achieved for the 511 keV photon-peak energy of ^22^Na. By using ^137^Cs, the averaged energy resolution was 3.73% at 662 keV of photon-peak energy. By applying the calibration for each anode pixel, the system achieved a spatial resolution of 3.07 mm, which was the FWHM of the profile of the point image. However, the timing resolution was not stated in the publication.

Several Kromek CZT sensors grown by the traveling heater method (THM) was presented in [165], which showed relatively good material uniformity and charge transport properties. A 40 × 40 × 15 mm^3^ large volume of 20 × 20 pixelated detectors had achieved an energy resolution of <2.5% at 662 keV at room temperature without correction. Another 11 × 11 pixelated detector with a volume of 22 × 22 × 15 mm^3^ achieved <1.5% energy resolution with over 50% of the pixel. After applying a conventional DOI correction, the energy resolution improved to 0.76%. Using the subpixel calibration resulted in an even better energy resolution of 0.67%.

Another way to use a CZT detector to improve the performance of a PET scanner was discussed in [166,173]. The technique, called Virtual-pinhole PET (VP-PET) imaging, uses one or more high-resolution detectors inserted into a traditional PET scanner with lower resolution detectors. In [166], the inserted module was a 3 × 3 pixelated CZT detector with 250 μm pixel size and 350 μm anode pixel pitch. The overall dimension of the detector was 40 × 40 × 15 mm^3^. The anodes were formed by titanium with a thickness of 100 nm while the cathode was produced by gold with a thickness of 125 nm. The 9-anode structure detector connected with the readout PCB through pogo-pin connectors. The authors paid special attention to the charge sharing (called double-pixel) detection during the characterization of their system. The detector achieved a 7% energy resolution at FWHM for a single pixel and 9% for the double-pixel photoelectric detections at 511 keV. The FWHM of the timing resolution was 30 ns for single pixel detection and of 35 ns for the double-pixel detection. The position resolution achieved was 350 μm FWHM in the *x*- and *y*- directions and 0.4 μm FWHM for DOI. In order to investigate the limitation caused by the spatial charge distribution, Monte Carlo (MC) simulations were performed. The simulation results showed an intrinsic spatial resolution of 170 μm FWHM for 511 keV high energy gamma rays. By applying interpolation for Compton-scattered events, the simulations showed that a CZT detector with 250 μm pixel size and 350 μm anode pixel pitch can achieve the highest energy resolution of 0.6% (FWHM) at 511 keV and a timing resolution of 2 ns (FWHM).

#### 6.2.2. Cross-Strip Electrodes

As mentioned previously, CZT detectors with the cross-strip electrode structure are attractive since the number of channels are significantly reduced, lowering the complexity of readout electronics when compared to a pixelated detector of the same active area. This feature is especially important when considering the fact that PET detector rings usually utilize hundreds of CZT detectors. Due to this advantage, intensive research efforts were taken not only at the detector level, but also at the system level.

Cross-strip electrode CZT detectors have been investigated in [167] and [174]. In [167], the authors deposited 16 anodes and five cathodes on each side of the CZT crystal with a volume of 20 × 16 × 0.9 mm^3^. The anodes, designed with a width of 0.9 mm on a 1 mm pitch, were arranged orthogonally with the cathode segments with a width of 3.9 mm on a 4 mm pitch. After applying a bias voltage of 500 V and a 300 keV energy threshold, the coincidence timing resolution was measured at 2.1 ns FWHM on the anode and 1.6 ns FWHM on the cathode and 2.6 ns FWHM with the setup consisting of two detectors and a ^68^Ge source in the middle of the two detectors. A simulation was performed by using the Monte Carlo package Penelope on 16 stacked layers of two detector modules. The stacked CZT detector had a volume of 16 × 16 × 40 mm^3^ and was divided into 2560 voxels in the simulation. The simulation results showed that the spatial resolution was less than 1 mm with implementation of DOI correction. In [168], a dual-panel positron PET camera for breast cancer imaging using Monte Carlo simulation was studied. The system employed two CZT panels with a dimension of 4 × 12 × 15 cm^3^. The cross-strip electrode pattern in the CZT detector enabled direct photoanode measurements, which provided accurate 3D photon interaction positions. The simulations showed that the system achieved photon sensitivity of around 32% for a point source in the center, 10% energy resolution at 511 keV and coincidence timing resolution of around 8 ns FWHM. As for the spatial resolution, the system achieved 1 mm (FWHM) intrinsic resolution in any space between the two panels with a 4 cm panel separation assuming a <2mm DOI resolution of the detector.

In order to improve the charge collection of the anode, another type of cross-strip pattern was intensively researched. In this type, there are additional steering electrodes placed between the anode strips. By applying an appropriate biasing voltage on the steering electrodes, the electric field near the anode electrodes bend towards them, thus enhancing their charge collection efficiency. In [169], a Small Animal PET System based on the cross-strip CZT detector was presented. The volume for the single CZT detector was 39 × 39 × 5 mm^3^. Thirty-nine anodes were deposited with 0.1 mm width and 1.0 mm pitch and the eight cathodes were arranged orthogonally with 4.9 mm width and 5.0 mm pitch on the other side. Thirty-eight steering electrodes were placed between the anodes with a width of 0.2 mm. The signals from both anode and cathode were read by RENA-3 (Readout Electronics for Nuclear Applications) ASICs. In this system, two methods had been used to determine the DOI resolution. One was called the charge-drift-time method, in which the DOI information was calculated based on the time difference of the signal from anode and cathode by considering the electron drift velocity. The other was the cathode-to-anode ratio method. The ratio of the pulse height between the cathode and anode was used to calculate the DOI position. The ratio ranged from 0 for the interaction at the anode to nearly 1 for the interaction at the cathode and was almost fully dependent on the position of interaction. Both methods showed that the system achieved a DOI accuracy <1 mm. By biasing the cathodes at –500 V and the steering electrodes at –136 V, the system achieved an energy resolution of 2% at 662 keV and 2.2% at 511 keV. 

Further research on cross-strip CZT detectors was conducted in [163]. The detectors’ ability to provide the 3D position of the photon interaction had been investigated for PET application. The detectors were fabricated by using 40 × 40 × 5 mm^3^ monolithic CZT crystals with 38 anodes (0.1 mm width, 1.0 mm pitch) and seven cathodes (5.4 mm width, 5.5 mm pitch). Similarly, the steering strips with a width of 0.2 mm were arranged between the anodes, and signals from the anodes and cathodes were read by the RENA-3 ASICs. After biasing the cathode at –500 V and the steering electrodes at –46 V, the performance parameters of the prototype system were evaluated by placing a ^22^Na point source in the middle of the two detectors. The prototype system achieved an energy resolution of 3.9 ± 0.19% FWHM at 511 keV. The DOI information was acquired by measuring the ratio of signal amplitudes from the anodes and cathodes. The point spread function was measured to be 0.78 ± 0.10 mm FWHM. The study also showed the function of steering electrodes. By biasing the steering electrodes at –100 V lower than the reference voltage on the anodes, the anodes achieved full charge collection. 

The first characterization results of a newly developed prototype of a whole PET system based on CZT detectors were showed in [170,171,175]. Each single CZT detector had 39 anode strips (0.1 mm width, 1.0 mm pitch), 38 steering electrode strips (0.4 mm) placed between the anode strips and eight cathode strips (4.9 mm width, 5.0 mm pitch). Two 40 × 40 × 5 mm^3^ monolithic CZT detector were stacked to form a 40 × 40 × 10 mm^3^ CZT dual-module by using anode–cathode–cathode–anode (ACCA) stacking. The readout system was design by using RENA-3 ASICs. The prototype sub-system consisted of two detection panels facing opposite directions. Each panel had three CZT dual-module stacked detectors, which means the full sub-system included 12 single CZT detectors. The energy resolution of all 468 anode channels in the sub-system was characterized by using ^68^Ge and ^137^Cs radioactive isotopes. The measurements showed that the averaged energy resolution over all anodes was 7.35 ± 1.75% FWHM by considering the signal variation caused by the depth difference of the photon interaction.

A summary and comparison of CZT detectors is shown in Table 8. Both pixelated and cross-strip CZT detectors can provide excellent performance in terms of energy resolution and position resolution. An energy resolution as low as <1% is achievable, and the position resolution can be measured as low as 0.4mm. However, the timing resolution of CZT detectors lags behind other technologies with a range of nanoseconds to tens of nanoseconds. The poor timing performance is further discussed in Section 8.2.1. 

## 7. State-of-the-Art Systems

In real clinical applications, PET imaging systems are usually not utilized alone but with other imaging modalities like X-ray CT and MRI. The dual-modality imaging systems like PET/CT and PET/MRI acquire their images simultaneously in the same bed position, which help to reduce errors for image registration and image fusion, provide more diagnosis information and reduce the scanning time of patients. In addition, the imaging from one modality can be used for imaging reconstruction and correction in the other imaging modality. PET/CT systems have been widely used in many pre-clinical and clinical areas for decades, while PET/MRI systems were just released within the past several years due to working limitations of PMTs in the magnetic field. Thanks to the technological advancements of solid-state sensors like APD, SiPM, and dSiPM, many PET/MRI imaging systems were designed and developed.

### 7.1. PET/CT Systems 

There are several medical device manufacturers providing PET/CT systems. Table 9 lists the state-of-the-art PET/CT system from these several medical device manufacturers. In this section, only the design and performance of the PET system will be compared and discussed. The system performance parameters such as spatial resolution, timing resolution, and energy resolution values are reported as FWHM.

By using a dSiPM (DPC series), the Philips Vereos PET/CT offered the first commercial digital PET system with enhanced ToF performance [176]. The basic detector consisted of DPCs and arrays of LYSO scintillators of size 4 × 4 × 19 mm^3^ coupled on DPCs through direct 1:1 coupling. Due to the significantly better performance of Philips DPC technology, the overall system achieved a spatial resolution of 4.1 mm, a timing resolution of 325 ps and an energy resolution of 11.1% [177].

Discovery MI PET/CT is the latest PET/CT system from GE. The detection ring of the PET system is made of tens of Lightburst Digital Detectors, which combines a small lutetium-based scintillator (LBS) crystal array with a SiPM [178]. The 4 × 6 mm^2^ SiPM pixels were arranged in a 2 × 3 array to form a SiPM device with a large active area of 12.6 × 12.6 mm^2^ and a small dead area among the pixels (as low as to 250 µm). The photosensitive area of the SiPM device was then coupled to a 4 × 3 array of LBS crystals of size 3.95 × 5.3 × 25 mm^3^. The output signals of the SiPM were processed by a custom ASIC to enhance the integrity of the shape of the SiPM output and lower the power consumption of the module. Based on the modular design concept, the Lightburst Digital Detector can be assembled onto the gantry with different sizes to build a scalable PET system. The system measurement results showed a typical spatial resolution of 4.2 mm, a timing resolution of 385 ps and an energy resolution of 9.4% [179].

The PET system of Biograph Vision PET/CT is the fastest ToF PET currently in the market [180], achieving a CRT of 214 ps [181]. A so-called ultra-dynamic range (UDR) detector was designed for this system. An array of fast LSO scintillator of size 3.2 × 3.2 × 20 mm^3^ was coupled on the SiPM to form the UDR detector with a 100% coverage. With the smaller scintillator size and the fastest ToF performance, the Biograph Vision PET/CT achieved a spatial resolution as small as 3.6 mm. However, a small scintillator size means the Biograph Vision PET/CT needs more crystals than other systems to achieve the same FoV, leading to increased complexity in the fabrication and assembly of the scintillators. The energy resolution of the system was measured to be 9% [182].

Interestingly, the newest PET/CT system from Toshiba (now part of Canon Group) called the Celesteion™ PUREViSION Edition PET/CT system [183], is still based on PMT technology. The PET detector was designed for a large-bore PET. The detector was made of PMTs with mixed sizes and lutetium-based scintillators (LBS). With optimal use of LBS and a unique mixed PMT configuration, the system achieved a spatial resolution of 5.1 mm, a timing resolution of 394 ps, and an energy resolution of 11.2%, which were comparable to PET systems based on solid-state sensor technology like SiPM and dSiPM.

**Table 9:** Comparison of PET performance in the state-of-the-art PET/CT systems.

Reference	[176,177]	[178,179]	[180,181]	[183]
Manufacturer	Philips	GE	Siemens	Canon
Model Name	Vereos Digital PET/CT	Discovery MI PET/CT	Biograph Vision PET/CT	Celesteion™ PUREViSION Edition PET/CT
Scintillator Material	LYSO	LBS	LSO	LBS
Scintillator Size (mm^3^)	4 × 4 × 19	4.0 × 5.3 × 25	3.2 × 3.2 × 20	4 × 4 (Length Unknown)
Sensor	dSiPM	SiPM	SiPM	PMT
Spatial Resolution (mm)	4.1	4.2	3.6	5.1
Timing Resolution (ps)	325	385	214	394
Energy Resolution (%)	11.1	9.4	9	11.2

### 7.2. PET/MRI Systems 

Due to the incompatibility with magnetic fields, the PMT-based PET systems can only be combined with MRI systems using extra shielding techniques. Unlike the PMT, solid-state sensors are compatible with the magnetic field. Therefore, PET systems based on solid-state sensors can be directly integrated with the MRI system, which accelerated the development of the hybrid PET/MRI image modality. There are many advantages for this hybrid system. First, the PET/MRI system can take the advantages of both image modalities; PET can provide a functional image to show the metabolic process while MRI shows the excellent soft-tissue contrast. Second, PET and MRI images acquired simultaneously from the same patient position have higher quality when compared to the fused PET and MRI images taken from individual PET and MRI systems at different times. Third, compared to the PET/CT system, the PET/MRI system can achieve a reduced radiation exposure since there is no X-ray involved in the system. Table 10 shows a list of state-of-the-art PET/MRI systems in the commercial market. Similar to the previous section, only the design and performance of the PET system will be compared and discussed, and the spatial resolution, timing resolution, and energy resolution values are reported as FWHM.

The Ingenuity TF PET/MRI system is a sequential hybrid PET/MRI system for whole-body imaging, which consists of two standalone systems - a Philips Astonish ToF PET and a Philips Achieva 3T MRI system [11]. In this design, the PET system and MRI system are installed face-to-face separately and a turntable between the PET and MRI is employed to move the patient into the different systems to acquire the images. The PET system in this hybrid system is based on PMT technology. In order to avoid the mutual system interference, especially the magnetic field interference on the PMTs, a magnetic shielding was introduced in the PET gantry design. The array of LYSO of size 4 × 4 × 22 mm^3^ was coupled on the PMT module to form a basic detector. The overall PET system achieved a spatial resolution of 4.7 mm, a timing resolution of 550 ps, and an energy resolution of <13% [12,184,185].

The SIGNA™ PET/MRI hybrid system from GE uses the same ToF PET as in the Discovery MI PET/CT [13]. As mentioned in the previous section, the PET system is developed based on SiPM technology, which is compatible with the magnetic field and there is no requirement for a shielding design for the PET system. Compared to the Ingenuity TF PET/MR system, another advantage of the SIGNA™ PET/MR hybrid system is that it is a concurrent system, acquiring the PET image and MRI image at the same patient position at the same time, resulting in a reduced scan time and an improved accuracy for PET and MRI image co-registration and fusion.

The PET system in Siemens Biograph mMR system is based on APD technology [14]. There is no shielding requirement for the PET system since the APD’s performance is not affected by the magnetic field. Even though the PET system cannot use the ToF techniques due to the poor timing performance of APDs, the system still achieved a spatial resolution of 4.6 mm when using LSO scintillators of size 4 × 4 × 20 mm^3^.

## 8. Research Challenges and Conclusions

Due to the need for a scintillator to convert gamma rays into visible light, the photosensors PMT, APD, analog SiPM, and dSiPM can be categorized as indirect-type sensors. In this category, it is clear that PMTs and APDs have been replaced by SPAD-based sensors—analog SiPM and dSiPM—in PET applications due to their compatibility with magnetic fields, excellent timing resolution, low operating voltage and highly integrated readout and signal processing system. The investigation and research on SiPMs will attract more attention and interest. The employment of ToF techniques further improve the SNR and effective sensitivity of the PET system, resulting in an improved image quality. Many research groups and companies have managed to achieve good performances with analog SiPMs, readout ASICs for analog SiPMs and dSiPMs and their application in the PET systems. However, there are still some existing research challenges.

### 8.1. Research Challenges for SiPMs 

#### 8.1.1. Improving the PDE of CMOS SiPMs

Although SiPMs can achieve good performance in the HV CMOS and CIS technologies, SiPMs implemented in standard CMOS technology are preferred due to their lower cost and the potential benefits from the integration of the SiPM and TDCs on one chip. This is especially important because thousands of dSiPMs will be needed to build a detector ring for a human-sized PET system. However, unlike the CIS technology, in which the passivation layer will be removed on the active sensing area, the passivation layer on the top of the sensing area of the SiPMs implemented in standard CMOS process still exists after fabrication. Therefore, a large number of incident photons will be reflected and absorbed when passing through the thick passivation layer and internal dielectric oxide layers, resulting in only a small portion of the incident photons arriving at the active sensing area of the SiPM. In addition, the depletion region of the p-n junction of SPADs (SiPM pixel) is usually thin, which leads to a lower photon detection probability in the depletion region. Due to these two main reasons, SiPMs implemented in the standard CMOS technology suffer from low PDE performance. In order to achieve a better PDE, research efforts are needed to address this challenge. 

First, proper post-fabrication techniques can be used to remove or thin the passivation layer to increase light transmission [186]. Further surface treatments like depositing an anti-reflection coating to reduce the surface reflection or producing micro-lens arrays to focus the light onto the active area can be used to boost the light transmission. A SiPM with the micro-lens array to improve the PDE was reported in [187]. By depositing micro-lenses on the surface of the SiPM, the highest effective FF was 84% while the native FF was 28%. However, these post-fabrication steps will inevitably increase the fabrication cost. Furthermore, the use of micro-lens can still be ineffective in dealing with the light exiting from scintillators under wide angles in PET applications.

Second, SiPMs with a 3D structure show great potential to achieve high PDE due to their high FF. In the 3D structure, the SiPM is implemented in the top tier of the silicon wafer while the other signal conditioning and processing circuits such as quench and reset circuits, counters and TDCs, are implemented in the second tier of the wafer. Signals between the SiPM on the top tier and the circuits on the second tier are connected by TSVs (through-silicon vias). The TSV bonding technology has been successfully realized in a commercial vertically integrated sensor system. The 6 × 6 mm^2^ SiPM sensors available from SensL (now part of ON Semiconductor) can achieve a FF of greater than 90% [188]. As discussed in Section 5.3.3, the SPAD junctions show good performance by employing less-scaled technologies, which have lower doping concentration resulting in a thicker SCR. This results in a higher PDE and a lower DCR caused by tunneling. On the other hand, the smaller, advanced CMOS technologies are preferred for the signal conditioning and processing circuits like the TDCs and counters so that they can achieve shorter cell delay times, higher operational frequency, and lower power consumption. By using the 3D structure, the top and bottom tiers can be optimized and fabricated in their separate processes. Thus, there is no need to compromise the performance between the SiPMs and the circuits for signal conditioning and processing when they are manufactured in the same process technology, thus achieving the highest performance for the overall system.

#### 8.1.2. Lowering the Dark Noise of SiPM Pixels (SPADs)

As discussed in Section 5.2, several carrier generation mechanisms such as generation-recombination, band-to-band tunneling (BTBT) and trap-assisted tunneling (TAT) are responsible for the DCR. The generation-recombination centers induced by the crystal defects and/or the impurities from the material and fabrication process create discrete generation-recombination energy levels in the forbidden energy bandgap, thus trapping and releasing the free carriers according to the Shockley-Read-Hall (SRH) mechanism. If the thermally generated free carriers occur within the diffusion length of the depletion region of the SPAD, a false dark noise pulse might potentially be triggered. Since the SRH generation rate increases exponentially with the increase of the temperature, the dark noise due to the generation-recombination mechanism can be reduced by lowering the temperature using cooling methods like forced air and/or thermoelectric cooling. 

Tunneling including BTBT and TAT are another important mechanism for dark noise. Band-to-band tunneling occurs when there is a high electric field across a strongly reverse-biased p-n junction, resulting in a significant flow of electrons from p-side the valence band (VB) to the n-side conduction band (CB). The trap centers within the forbidden band due to defects and impurities assist the tunneling from the VB to CB, giving the process the name of trap-assisted tunneling. The DCR by tunneling becomes dominant in SPADs fabricated in DSM CMOS technology due to the decreased depletion width and abrupt doping profiles [189]. In order to reduce tunneling, the electric field within the junction should be lowered by decreasing the biasing voltage, but doing so inevitably lowers the PDE as well.

In addition to thermal generation and tunneling, another source of the DCR of SPADs is after pulsing (AP). In order to minimize the AP, the bias for the SPAD should stay below breakdown voltage for a sufficient amount of time after quenching to ensure that the trapped carriers can be released without triggering after pulses, which can be implemented by an AQR circuit. However, an increased hold-off time prolongs the dead time of SPAD, thus reducing its counting rate. Moreover, when designing the AQR, one should try to minimize the input capacitance of the circuits, which is the load capacitor of the SPAD, thus lowering the charge generation during the avalanche process and ultimately lowering the trapping probability of carriers. Another important point worth noting is that the temperature characteristic of AP shows an opposite trend to that of thermal DCR. AP becomes the dominant source of DCR when SPADs are cooled in an effort to reduce the thermal DCR, since the trapping lifetimes increase at lower temperatures [62,63]. Therefore, an optimal operating temperature can be found from a compromise between the thermally generated dark noise and the AP.

#### 8.1.3. Reducing the Timing Jitter

In PET applications, the timing information for the detectors is processed by the coincidence unit to identify two coincident gamma rays that come from one true annihilation event. The increased accuracy of detecting the time difference between two coincident gamma rays lower the uncertainty of the occurred event along the LoR. Therefore, higher time resolution means higher SNR of the PET image. With improved time jitter below 500 ps, it is possible to use the ToF technique in PET to obtain great performance enhancements when compared to the conventional PET. A “10 ps challenge” which aims to achieve a CRT of 10 ps FWHM for ToF application was set up recently [190]. With a 10-ps CRT, a high-sensitivity, reconstruction-less PET scanner would achieve reduced scanning time, scanning cost and the radiation dose. In order to achieve this challenge, one of the key issues is to reduce the timing performance of the photosensor in the PET detector. 

The sources of the timing jitter in a SiPM-based detector can be generally categorized into four types: jitter from the SPAD junction of the SiPM pixel, jitter from the comparator, jitter from the TDC and jitter from the connections between different blocks on the timing signal chain. For the SPAD junction, there exists an inherent timing jitter due to the variation of delays between photon absorption and the build-up time of the avalanche pulse [191]. Typically, SiPMs based on shallow and thin depletion regions can achieve the best timing performance. Junctions biased at a high excess voltage, which have a high electric field in the active region, achieve a lower timing jitter because the avalanche build-up time becomes more certain statistically [192]. However, the high excess voltage will increase the DCR.

The amplitude of the output signal of a SiPM varies due to the stochastic uncertainty of the avalanche process. If a general leading-edge discriminator (LED) with a fixed threshold is used, the conversion process from the analog output of a SiPM to a digital pulse inevitably gives rise to timing jitter. One possible solution to minimize this jitter is to use a constant fraction discriminator (CFD), which keeps a constant timing output even when the amplitude of the SiPM’s output signals vary. CFD requires more silicon space due to its complexity, thus lowering the FF of the SiPM, but the space requirement might not be an issue for a 3D dSiPM.

The TDC also contributes timing jitter to the whole system due to the different noise sources (e.g., jitter from the delay lines and reference clock) and performance differences between delay cells because of the parasitic mismatch, and process, voltage, and temperature (PVT) variations. By down-scaling the CMOS technology, the digital units in the TDC can achieve less jitter, less delay and high operating frequency. The 3D dSiPM is a good solution for this issue since the TDCs and SiPM can be implement in their preferred CMOS technologies. However, dealing with a large number of connections between the SiPM layer and circuit layer is also challenging. In addition, special placement and routing of delay cells and dummy cells can be used to ensure the matched parasitics of the delay cells, and a delay-locked loop can minimize the influence of PVT variations in many TDC structures.

The timing jitter from the connections on the signal chain can be minimized due to the integration of the SiPM with TDCs in one chip. The one-chip integration can eliminate many off-chip influences such as I/O pads, bonding wires, and on-board matching issues, which exists in a system using separate SiPM and TDC chips.

### 8.2. Research Challenges for CZT Detectors

A timing jitter in the range of tens of nanosecond unfortunately means that CZT detectors are currently not suitable for ToF applications. However, the excellent position resolution (<1 mm FWHM) and energy resolution (<3% FWHM @ 511 keV) make CZT sensors perfect detectors for small-sized PET systems such as those for small animals, in which the ToF technique is not so crucial due to the small size of the PET ring. Another advantage of the CZT detector is that the position resolution can be reduced by controlling the pattern and size of the readout electrodes. In comparison, the position resolution of a scintillator-based indirect-conversion detector is highly dependent on the size of the individual crystals in the scintillator block. Generally, the individual crystals need to be fabricated to roughly the size of the required resolution, which can be observed in Table 9 and Table 10. The requirement for the small-sized crystals leads to an increase of the fabrication cost and a reduced yield due to the difficulty to control material defects. In addition, the DOI information can be easily derived from the signal amplitudes ratio and/ or the signal timing difference from both the anode and cathode, giving more accurate positions of interaction and LORs. However, there are still some research challenges of CZT detectors in PET applications.

#### 8.2.1. Improving the Timing Performance

As a direct-conversion detector, the signals for both electrodes are generated due to the drift of charge carriers triggered by the impact ionization due to the high energy gamma ray. Since the sensitivity of the electrode varies spatially, the output signal carries a relatively high temporal variance. That is why the CZT detectors suffer more from time jitter and time walk, degrading the CRT into the range of tens of nanoseconds. One possible solution is to reduce the distance between the two electrodes in order to reduce the drift time of the charge carriers. In [167], it was demonstrated that one CZT detector with a thickness of 0.9 mm was able to achieve a CRT of 2.6 ns FWHM. Even though this performance is still worse than the timing performance in scintillator-based indirect-conversion systems, the timing performance from this thin CZT detector can still help to reject random events. However, by using a thin CZT detector, more detectors are needed to build a detector ring of the same size when compared to a system with thick CZT detectors. This inevitably increases the cost of fabrication of the CZT detectors and readout electronics due to the increased signal channels.

#### 8.2.2. Processing Small Amplitude Readout Signals

Compared to the commonly used L(Y)SO scintillator material (atomic number of ~66 and density of ~7.4 g/cm^3^) in PET applications, CZT has a relatively low atomic number and density (average atomic number of 49.1 for Cd_0.9_Zn_0.1_T with a density of 5.78 g/cm^3^). As a result, the incident gamma rays are predominately Compton scattered rather than photoelectrically absorbed. A Monte Carlo simulation of a small animal PET system based on a cross-strip CZT shows that only 24.9% of photons undergo photoelectric absorption while the rest of photons deposit their energy to the CZT material through Compton scattering [193]. The simulations showed that the 511 keV incident gamma photon would deposit the energy to 2.2 voxels on average due to the Compton scattering. Since only a portion of the 511 keV energy is deposited due to the Compton scattering dominance, the amplitude of the output signal is a relatively small. In order to process the small-amplitude signal, charge-sensitive readout electronics with very low noise are required to achieve a reasonable SNR.

#### 8.2.3. Managing a Large Number of Electrodes

According to the signal generation mechanism of CZT detectors, very good performance can be achieved by controlling pattern and reducing the size of the electrodes. However, this leads to an increased number of cathode and anode channels, requiring a large number of readout channels to process the analog signal with relatively small amplitude. Unlike the dSiPM, where the sensors and the readout electronics can be implemented on the same wafer or on the same package, the CZT detector requires separate readout electronics. The readout in a lot of PET applications using the CZT detector has thus relied on an ASIC called RENA-3—a mixed signal ASIC with 36 channels of low-noise, self-resetting, charge-sensitive preamplifiers and shapers developed for position-sensitive solid-state detectors [194]. Even though this specifically designed ASIC can provide 36 readout channels, the integration of CZT detectors can still be challenging in terms of the cost of the ASIC, the robustness of the interconnections between the CZT detector and readout ASIC, data acquisition and transmission bandwidth. Crosstalk between the electrodes also presents a challenge. When a gamma ray hits the edge of one electrode or within the gap between two electrodes, charge sharing can occur. Because of this effect, the involved electrodes will all generate output signals, but with a relatively small amplitude when compared with that of a single-electrode interaction. However, even in a single-electrode interaction, the neighboring electrodes still generate small transient signals. If these effects are not properly processed, degradation of the PDE, spatial resolution and energy resolution of CZT detectors can be expected. In [162], the authors carefully studied the charge sharing effect and analyzed the transient signals by simulation through Geant4 Application for Tomographic Emission (GATE)—a numerical simulation platform for radiotherapy and medical imaging. The simulations showed that a CZT detector with dimensions of 20 × 20 × 5 mm^3^ achieved an SNR of ~17 and a subpixel spatial resolution of 30 µm by using the charge sharing method and of 250 µm by using transient signal analysis methods. 

### 8.3. Conclusions

In this paper, we presented a comprehensive review on four types of sensors for PET applications. Starting with the introduction of the physics of PET, TOF techniques for PET applications and the structure of the general PET detector, four types of sensors - PMTs, APDs, SiPMs and CZT detectors - were studied. For each type of sensor, we introduced their structures, operational principles, key performance parameters and their PET applications. An overall comparison between PMT, APD, SiPM, dSiPM, and CZT detectors is shown in Table 11. Among these four types of sensors, we focused on the two most promising type—SiPMs and CZT detectors. We also studied and compared the state-of-the-art commercial clinical PET systems which have trended towards using SiPM technology. SiPMs have been employed in all kinds of PET systems—preclinical small-animal PET systems, clinical human body PET/CT systems, and PET/MRI systems due to their excellent performance like high timing resolution, low operation voltage and features such as compactness and immunity to the magnetic field. However, efforts are still needed to improve the performance of SiPMs for lower noise, higher PDE, and even better timing resolution. It is predicted that dSiPMs might overtake analog SiPMs in future PET systems as integration efforts improve owing to progress in the packaging, interconnection, assembly, and fabrication of the semiconductor devices. Although CZT detectors showed excellent position and energy resolutions, their PET applications are limited to small-sized preclinical PET systems due to their poor timing resolution performance. Thus, more research is required for CZT-based detectors to be applied in larger PET systems.

## Figures and Tables

**Table 1:** Some commercial/tested PMTs for PET applications.

Reference, Year	Sensors	Crystal	Crystal Size (mm^3^)	CRT FWHM (ps)	Energy Resolution FWHM (%) ^a^
[34], 2019	Hamamatsu R1548	LGSO	2.45 × 5 × 15 (9 × 10 Array)	-	13.1
[31], 2013 [33], 2015	-	LYSO	2.35 × 2.35 × 15.2 (16 × 16 Array)	473	11.2
[30] ^b^, 2013	Hamamatsu R9800	LYSO	4 × 4 × 10(Single)	200.5	11.1
[30] ^b^, 2013	Hamamatsu R11194	LYSO	4 × 4 × 10 (Single)	227.8	11.8
[32], 2009	Hamamatsu R9779	LYSO	4 × 4 × 20 (13 × 13 Array)	432	-
[32], 2009	Photonis XP1912	LYSO	1.4 × 1.4 × 10 (13 × 13 Array)	551	-
[35] ^c^, 2011	Hamamatsu R9800	LYSO	4 × 4 × 10 (Single)	198 254	10.6 10.8
[29] ^d^, 2008	Hamamatsu PS-PMT R8400-00-M64 MOD	LYSO	2.9 × 2.9 × 20 (16 × 16 Array)	505	10.9

^a^ Energy resolution in the center of the PMT. ^b^ Average results are from the measurements of seven PMTs. ^c^ Results are from two different PMTs (R9800). ^d^ Average value.

**Table 2:** Some commercial/tested APDs for PET applications.

Reference, Year	Sensors	Crystal	Crystal Size (mm^3^)	CRT FWHM (ns)	Energy Resolution @511 keV FWHM (%)	Spatial Resolution FWHM (mm)
[38], 2005	Hamamatsu S8664-55 (2 × 2 Array)	LSO	2 × 2 × 20 (9 × 9 Array)	2.47	20.9	2
[39], 2008	Hamamatsu S8550	LSO	2 × 2 × 15 (Four Crystals)	2.15 ± 0.11	12.8 ± 0.6	-
[40], 2007	LabPET detector	LYSO/LGSO phoswich pair	2 × 2 × 10/12 (Single of Each)	6.6 ^b^ 10.7 ^c^	24 ± 6 (LYSO) 25 ± 6 (LGSO)	1.3–1.4
[41], 2015	LabPET II detector (two 4 × 8 arrays)	LYSO	1.2 × 1.2 × 12 (8 × 8 Array)	3.6 ± 0.3	20 ± 1	0.81 ± 0.04
[42], 2013	RMD PSAPD detector	LYSO	0.9 × 0.9 × 1 (8 × 8 Array)	15.7 ± 0.2	10.62 ± 0.04	0.84 ± 0.02
[43], 2011	Hamamatsu S8550-02	LYSO:Ce	21.4–23.5 × 18.5 × 10 (Monolithic) ^a^	27	13.2	2.12–2.64

^a^ Double-layer trapezoidal crystals varying in the transverse direction (21.4/22.4 for one, 22.5/23.5 for the other). ^b^ LYSO/LYSO coincidence. ^c^ LGSO/LGSO coincidence.

**Table 4:** Some commercial/tested analog SiPMs for PET applications.

Reference, Year	Sensors	Crystal	Crystal Size (mm^3^)	Readout & DAQ Electronics	Sensitivity (%) CRT (ps)DOI FWHM (mm)	Energy Resolution FWHM (%)	Spatial Resolution FWHM (mm)	Applications
2016, [113] ^a^	SensL (MicroFB-30035-SMT) 12 × 12 Array	LYSO (Three-Layer Staggered)	1.5 × 1.5 × 6 (35 × 35 Array, Top 36 × 36 Array, Middle 37 × 37 Array, Bottom)	Diode-Based Readout, Customized ADC Board	- - 6	11.8 @ 511 keV (Top) 9.6 @ 511 keV (Middle) 10.2 @ 511 keV (Bottom) (21 °C)	-	MINDView Brain PET Insert
	SensL (MicroFC-30035-SMT) 12 × 12 Array	LYSO	50 × 50 × 20 (Monolithic)	Resistive Readout, Customized ADC Board	- - 2	17 @ 511 keV (21 °C)	1.5	
2018, [114] ^b^	SensL (MINDView-Series) 12 × 12 Array	LYSO	50 × 50 × 20 (Monolithic)	Customized Readout and ADC Board	7 ^c^ - 4 ± 1	17.5 ± 1.5 @ 511 keV (27 °C) ^d^	1.7 (CFOV) <2 (within the 120 mm Diameter of the Center) ^e^	MINDView Brain PET Insert
2017, [118] ^a^	AdvanSiD (NUV-SiPMs) 4 × 8 Array	LYSO (Dual-Layer Staggered)	3.3 × 3.3 × 8 (7 × 7 Array, Top) 3.3 × 3.3 × 12 (8 × 8 Array, Bottom)	TRIROC ASIC (64 Channels)	7 - -	16 @ 511 keV (Top) 18 @ 511 keV (Bottom)	-	PET/MRI/EEG TRIMAGE System
2019, [119] ^a^	AdvanSiD (NUV-SiPMs) 4 × 8 Array	LYSO:Ce (Dual-Layer Staggered)	3.3 × 3.3 × 8 (7 × 7 Array, top) 3.3 × 3.3 × 12 (8 × 8 Array, Bottom)	TRIROC ASIC (64 Channels)	- 529 (Top)/501 (Bottom) -	22 @ 511 keV (Top) 20 @ 511 keV (Bottom)	-	PET/MRI/EEG TRIMAGE System
2018, [122] ^a^	SensL (J-Series) 2 × 2 Array	LYSO	2.76 × 2.76 × 18.1 (6 × 7 Array)	-	- 409 ± 39 -	11.7 ± 1.5 @ 511 keV	-	Total-body Human EXPLORER: PET/CT System
2016, [125] ^b^	SensL (C-Series) 12 × 12 Array	LYSO (Pyramidal Shape)	Entrance Surface: 48 × 48 mm^2^ Exit Surface: 50 × 50 mm^2^ Thickness: 10 mm (Monolithic)	Customized ADC board (66 Channels)	2.8 ^f^ - 2	15 ± 2 @ 511 keV (22 °C) ~14 @ 511 keV (15 °C) ^d^	~1 (whole FOV Range) ^g^	Small-Animal PET Insert

^a^ The performances for this reference is performance of the PET detector. ^b^ The performances for this reference is performance of the PET system. ^c^ The sensitivity was measured with an energy window of 350–650 keV at the center of the FOV by moving the 1 mm ^22^Na point source along the axial direction with a 0.5 mm step. ^d^ The energy resolution was the average value for the entire system. ^e^ System spatial resolution, measured by moving a small ^22^Na point source (0.25 mm diameter) along the radial direction at three positions along the axial direction: the center of FOV (CFOV), 0.25 and 0.375 of the axial axis. f The sensitivity was measured with an energy window of 358–664 keV by using a 0.25 mm ^22^Na (NEMA standard) point source. ^g^ System spatial resolution, measured by placing a 0.25 mm ^22^Na (NEMA standard) point source at different radial distances (from 0 mm to 25 mm with a step of 5 mm) at the center of axial FOV.

**Table 5:** Digital Photon Counter (DPC) dSiPMs for PET detector applications.

Reference, Year	Sensors	Crystal	Crystal Size (mm^3^)	Energy Window (keV)	Trigger Scheme	CRT FWHM (ps)	Energy Resolution FWHM (%)	Spatial Resolution FWHM (mm)
[137], 2015	DPC-3200-22-44	LYSO	2 × 2 × 6 (Single)	FWTM ^a^	-	171	12.6	-
		GAGG	2 × 2 × 6 (Single)	FWTM ^a^	-	310	8.5	-
		GAGG	3.2 × 3.8775 × 8 (8 × 8 Array)	FWTM ^a^	-	619	9.2	-
[144], 2016	DPC-3200-22-44	LYSO	32 × 32 × 5 (Monolithic)	400–650	2	529	23	0.6 ^b^
[141], 2016	DPC-3200-22-44	LYSO:Ce	32 × 32 × 22 (Monolithic)	FWTM ^a^	1	147	10.2	1.1 ^c^
[142], 2016	DPC-3200-22-44	LYSO:Ce	32 × 32 × 22 (Monolithic)	FWTM ^a^	1	214	10.2	1.7 ^c^
[136], 2013	DPC-6400-44-22	LSO:Ce,Ca	24 × 24 × 10 (6 × 6 Array)	450–570	2	-	12.8	0.97 ^d^

^a^ FWTM (full width at tenth maximum) of the 511 keV peak was applied. ^b^ Measured by using 0.4 mm diameter of gamma pencil beam (point ^22^Na sources in a collimator with 0.4 mm aperture). Mean nearest neighbor algorithm combined with DOI decoding was used to achieve this spatial resolution. ^c^ Measured by using 0.5 mm diameter of gamma pencil beam (point ^22^Na sources with a 0.5 mm diameter in a collimator). Maximum-likelihood estimation method was used. ^d^ Measured by using 0.5 mm diameter ^22^Na sources in a collimator.

**Table 6:** Digital Photon Counter (DPC) dSiPMs for PET system applications.

Reference, Year	Sensors	Crystal	Crystal Size (mm^3^)	# of Detectors	Energy Window (keV)	Trigger Scheme	CRT FWHM (ps)	Energy Resolution FWHM (%)	Spatial Resolution FWHM (mm)
[135], 2012	DPC-3200-22-44	LYSO	4 × 4 × 22 (8 × 8 Array)	10	440–660	-	266	10.7	~2.4 ^a^
[139], 2016	DPC-3200-22-44	LYSO	1.85 × 1.85 × 10 (16 × 16 Array)	12	-	-	298	-	~1.6 (CFOV) ^b^
[140], 2014	DPC-3200-22-44	LYSO	32 × 32 × 2 (Monolithic)	4 ^c^	400–650		680	18	0.7 ^d^
[143], 2018	DPC-3200-22-44	LYSO:Ce	32 × 32 × 22 (Monolithic)	32	FWTM	1	212	10.2	2.9 (CFOV) ^e^
[146], 2017	DPC-3200-22 (4 × 4 array)	LYSO	0.93 × 0.93 × 12 (30 × 30 Array)	10 ^f^	250–625 500–520	2 1	436.1 240.4	-	-
			4 × 4 × 10 (8 × 8 Array)		250–625 500–520	2 1	289.4 208.4	-	-
[148], 2018	DPC-3200-22 (4 × 4 array)	LYSO	0.93 × 0.93 × 12 (30 × 30 Array)	10	250–625	3	609	12.7	1.7 (CFOV) 2.5 (50 mm off the Center) ^g^

^a^ Measured using the NEMA NU-4 phantom and Mini Deluxe Derenzo phantom filled with 10 MBq FDG. Ordered subset expectation maximization (OSEM) algorithm with modeling of point spread function was used. ^b^ Measured by placing a hot rod phantom in the CFOV. Ordinary Poisson-OSEM (OP-OSEM) algorithm was used. ^c^ Four detectors formed a square shape. ^d^ Measured by using point ^22^Na sources (0.25 mm diameter) at the CFOV. 2D-OSEM algorithm was used. ^e^ Measured by using point ^22^Na sources (0.5 mm diameter) at the CFOV. 2D-filtered back-projection (FBP) algorithm was used. ^f^ Each module contained 2 × 3 detector stacks. ^g^ Measured by moving a point ^22^Na source along the radial direction at the center and 0.25 (22.5 mm) of the axial axis (following NEMA NU4-2008 standard). FBP algorithm was used.

**Table 7:** Summary and comparison of some dSiPMs.

	Reference, Year	[152], 2012	[151], 2014	[19], 2014	[153], 2014	[155], 2014	[154], 2017 ^c^
SPAD	Technology (CMOS)	130 nm	350 nm (HV)	130 nm (CIS)	130 nm	350 nm	150 nm
	No. of SPADs (in One Pixel)	1	16 × 26	24 × 30	1	1	2 × 2
	Breakdown Voltage (V)	14.4	–	–	–	28	18.8
	Cell Pitch (µm^2^)	50 × 50	30 × 30	–	48 × 48	–	60 × 60
	Active Area (µm, diameter)	8 (Circular)	19.7 × 16.5 (Rect.)	12.67 (Circular)	5 (Octagonal)	20 (Circular)	14 (Circular)
	Fill Factor (%)	2	21.2	35.7 ^b^	0.77	–	26.5
	Peak PDP (%)	25 @ 500 nm Vex = 1 V	30 @ ~420 nm Vex = 4 V	45 @ ~410 nm Vex = 1.5 V ^a^	30 @ 425 nm Vex = 1.5 V	43 @ ~420 nm Vex = 5 V ^a^	–
	Median DCR (Hz/µm^2^) @ R.T.	2.0 @ Vex = 1 V	250.0 @ Vex = 4 V ^a^	108.7 @ Vex = 1.5 V	26.2 @ Vex = 2.5 V	12.7 @ Vex = 5 V	44.2 @ Vex = 3 V
TDC	Clock Frequency (Hz)	280	50	100	–	–	–
	No. of TDCs (in one pixel)	1	48	2	1	1	0.5
	Resolution LSB (ps)	119	51.8	64.56	62.5	10	250
	Dynamic Range (ns)	100	–	261.59	64	160	–
	DNL (LSB)	±0.4 (Max)	1.97	–0.24 to +0.28	<4	–	–0.4 to +0.5
	INL (LSB)	±1.2 (Max)	2.39	–3.9 to +2.3	<8	–	–0.9 to +1.2 ^a^
	Counting Rate (MHz)	0.5	–	–	–	2.5	-
	No. of bits	10	19	12	10	–	16
	TDC/SPAD	1/1	3/26	2/720	1/1	1/1	1/8
System	Pixel Array	32 × 32	1 × 1	8 × 16	64 × 64	16 × 1	64 × 64
	Application	FLIM	PET	PET	FLIM	–	SNL

^a^ The values are read from the curves. ^b^ The size of TDC was included when calculating the fill factor. ^c^ The measurement results are based on imaging mode.

**Table 8:** Summary and comparison of some CZT detectors.

Reference, Year	Electrode Pattern	Anode Structure	Cathode Structure	Size (mm^3^)	Biasing Voltage (V)	Energy Resolution (% FWHM)	Timing Resolution (ns)	Position Resolution (mm FWHM)	Application
[165], 2018	Pixelated	20 × 20 Anode Array	Common Cathode	40 × 40 × 15	-	<2.5 @ 662 keV	-	-	SPECT
	Pixelated	11 × 11 Anode Array	Common Cathode	22 × 22 × 15	-	<1 @ 662 keV ^a^	-	-	SPECT
[164], 2016	Pixelated	8 × 8 Anode Array	Common Cathode	19.4 × 19.4 × 6	200–500	3.75 @ 511 keV 3.73 @ 662 keV	-	3.07	PET
[166], 2012	Pixelated	3 × 3 Anode Array	Common Cathode	20 × 20 × 5	1000	7 @ 511 keV ^b^ 9 @ 511 keV ^c^	30 ^b^ 35 ^c^	0.35 (*x*, *y*) 0.4 (*z*)	PET
[172], 2017	Cross-Strip	0.15 mm Width, 0.4 mm Pitch	1.9 mm Width, 2.0 mm Pitch	20 × 20 × 5	350	1.14 @ 662 keV	-	0.4	-
[171], 2017	Cross-Strip	0.1 mm Width, 1 mm Pitch (39 Anodes) 0.4 mm Width, 1 mm Pitch (38 Steering Electrodes)	4.9 mm Width, 5 mm Pitch (8 Cathodes)	40 × 40 × 5 ^d^	500	7.43 ± 1.02 @ 511 keV ^e^	37 ^e^	0.76	PET
[170], 2016	Cross-Strip	0.1 mm Width, 1 mm Pitch (39 Anodes) 0.4 mm Width, 1 mm Pitch (38 Steering Electrodes)	4.9 mm Width, 5 mm Pitch (8 Cathodes)	40 × 40 × 5 ^d^	500	7.35 ± 1.75 @ 511 keV ^e^	37 ^e^	0.76	PET
[168], 2010	Cross-Strip	0.05 mm Width, 1 mm Pitch	5 mm Pitch (Width Unknown)	40 × 40 × 5	-	3 @ 511 keV	8	1	PET
[169], 2008	Cross-Strip	0.1 mm Width, 1 mm Pitch (39 Anodes) 0.2 mm Width, 1 mm Pitch (38 Steering Electrodes)	4.9 mm Width, 5 mm Pitch (8 Cathodes)	39 × 39 × 5	500–1500	2 @ 662 keV 2.2 @ 511 keV	-	1	PET
[167], 2004	Cross-Strip	0.9 mm Width, 1 mm Pitch (16 Anodes)	3.9 mm Width, 4 mm Pitch (5 Cathodes)	20 × 16 × 0.9	500	-	2.6	1	-
[163], 2011	Cross-Strip	0.1 mm Width, 1 mm Pitch (38 Anodes) 0.2 mm Width, 1 mm Pitch (37 Steering Electrodes)	5.4 mm Width, 5.5 mm Pitch (7 Cathodes)	40 × 40 × 5	-	3.9 ± 0.19 @ 511 keV	-	0.78	PET

^a^ The value of energy resolution is shown after DOI correction. ^b^ The measurement results are based on single-pixel data. ^c^ The measurement results are based on double-pixel data. ^d^ The CZT detector is made of two layers of CZT blocks with a size of 40 × 40 × 5 mm^3^, resulting in an overall 40 × 40 × 10 mm^3^ CZT detector. ^e^ The measurement results of energy resolution and timing resolution are based on the whole system rather than on a single detector.

**Table 10:** Comparison of PET performance of state-of-the-art PET/ MRI systems.

Reference	[11,12]	[13]	[14]
Manufacturer	Philips	GE	Siemens
Model Name	Philips-Ingenuity TF PET/MRI Rystem	SIGNA™ PET/MRI Rystem	Biograph mMR System
Scintillator Material	LYSO	LBS	LSO
Scintillator Size (mm^3^)	4 × 4 × 22	4.0 × 5.3 × 25	4 × 4 × 20
Sensor	PMT	SiPM	APD
Spatial Resolution (mm)	4.7	4.57	4.6
Timing Resolution (ps)	550	385	N/A
Energy Resolution (%)	13	9.4	-

**Table 11:** Overall comparison of sensors for PET applications.

Type	PMT	APD	Analog SiPM	dSiPM	CZT
Conversion Type	Indirect	Indirect	Indirect	Indirect	Direct
Magnetic Field Compatibility	No	Yes	Yes	Yes	Yes
ToF Capability	Limited	No	Yes	Yes	No
Signal Readout	Analog	Analog	Analog	Digital	Analog
Operating Voltage	High	Low	Low	Low	High
Compactness	Low	Medium	Medium	High	Medium
Readout Electronics	Complex	Complex	Complex	Simple	Very complex
